# Catastrophic Degradation in Solid Oxide Fuel Cells Caused by Air Supply Interruption in Real‐World Operations: Fundamental Mechanisms and Mitigation Strategies

**DOI:** 10.1002/advs.202516807

**Published:** 2025-09-30

**Authors:** Haewon Seo, Ji‐eun Won, Wooseok Lee, Haneul Choi, Sun‐Young Park, Younghun Shin, Insung Lee, Tae Jin Lim, Kyeounghak Kim, Jongsup Hong, Hye Jung Chang, Kyung Joong Yoon

**Affiliations:** ^1^ Center for Hydrogen Energy Materials Korea Institute of Science and Technology Seoul 02792 Republic of Korea; ^2^ School of Mechanical Engineering Yonsei University Seoul 03722 Republic of Korea; ^3^ Clean Hydrogen Institute‐Research Support Department Korea Institute of Science and Technology Seoul 02792 Republic of Korea; ^4^ E&KOA Daejeon 34325 Republic of Korea; ^5^ Department of Chemical Engineering Hanyang University Seoul 04763 Republic of Korea; ^6^ Division of Nano Convergence KIST School University of Science and Technology Seoul 02792 Republic of Korea

**Keywords:** air supply, cathode, decomposition, solid oxide fuel cell, strontium

## Abstract

With the rapid market expansion of solid oxide fuel cells (SOFCs), real‐world operational incidents have unveiled various critical challenges. Malfunction of the gas supply system is among the most frequent and critical issues, severely damaging cells and stacks. However, the actual cause of this degradation remains completely unknown; existing explanations have been mostly speculative, and simple engineering‐based solutions have proven ineffective. The study reveals that air supply interruption induces irreversible chemical degradation of perovskite‐based electrode materials. Upon air stoppage, the continued electric current rapidly depletes oxygen, decreasing the oxygen partial pressure below the stability threshold of the state‐of‐the‐art (La,Sr)CoO_3–δ_ cathode. This triggers structural breakdown of the perovskite phase and initiates a cascade of harmful reactions with adjacent components and externally introduced impurities. Among the constituent elements, Sr is identified as a key driver of degradation, suggesting its elimination as a mitigation strategy. Then, by systematically tailoring the catalytic and electrical properties,a high‐performance Sr‐free cathode incorporating highly active nanocatalysts is developed. It outperformed the most advanced benchmark cathode, and more importantly, it demonstrated excellent durability upon severe air supply interruption with extreme oxygen depletion, offering a practically viable solution to a critical reliability challenge in commercial SOFC systems.

## Graphical abstract

Interrupting the air supply during the operation of solid oxide fuel cells leads to irreversible chemical degradation of the cathode materials, primarily driven by strontium at the A‐site of perovskite structures. Alternative strontium‐free materials effectively address this issue, ensuring high performance and reliable operation during operational incidents.

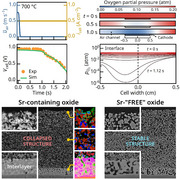

## 1 Introduction

With the escalating energy and environmental crises, there is a strong global effort to develop advanced energy technologies capable of replacing conventional fossil fuel‐based systems. Among these, solid oxide fuel cells (SOFCs) have emerged as a leading next‐generation solution, offering sustainable power generation with a low environmental impact. Operating at high temperatures, typically above 600 °C, SOFCs offer unique advantages, such as high efficiency, fuel flexibility, operational versatility, and high‐quality heat production. Extensive research over the past several decades has recently led to the successful commercialization of SOFC technology, and its market share is rapidly increasing across various sectors, including stationary power plants, distributed generation for buildings and residences, transportation, and military applications.^[^ ^1^ ^]^ However, reliability issues remain a significant challenge for SOFCs to economically compete with other conventional technologies.^[^ ^2^ ^]^ High‐temperature degradation in cells and stacks encompasses highly complex phenomena and has long been a central focus of SOFC research, leading to effective solutions for certain degradation issues. For example, the chemical interactions between the cathode and electrolyte have been suppressed by employing optimal diffusion barrier layers,^[^ ^3^ ^]^ and Cr poisoning has been mitigated by applying protective coatings to the metallic interconnects.^[^ ^4^ ^]^ Consequently, the stability of cells and stacks has improved substantially, with degradation rates below 1.0% kh^−1^ reported,^[^ ^5^ ^]^ which can make commercial products economically feasible.

Although reliable stability has been demonstrated under well‐controlled laboratory conditions, the real‐world operation of SOFC systems can present unexpected incidents that may lead to new types of serious problems. Thus, it is essential to thoroughly understand and prepare for potential issues arising from unforeseen situations to ensure genuinely reliable SOFC systems. One commonly encountered problem in SOFC system operation is the malfunction of gas supply systems. SOFC systems include two separate gas supply systems, one for fuel and the other for air. The effect of fuel supply interruption is relatively intuitive: an insufficient fuel supply increases the oxygen partial pressure on the anode side, which oxidizes metallic Ni to NiO. This oxidation causes a volume expansion within the porous composite electrode, resulting in mechanical failure. The mechanical damage caused by Ni re‐oxidation in the anode has been extensively studied.^[^ ^6^ ^]^ By contrast, the impact of air supply interruption on cell and stack stability has received relatively little attention from a scientific standpoint. In practice, the air blower is a vital balance‐of‐plant (BOP) component that operates under demanding conditions. Continuous excess airflow is required for thermal management during system operation,^[^ ^7^ ^]^ and this heavy workload frequently leads to air blower failure.^[^ ^8^ ^]^ In such events, significant performance degradation has been consistently observed across SOFC field operations worldwide.^[^ ^9^ ^]^ However, only a few studies have reported on this degradation phenomenon, with some attributing it to contact loss,^[^ ^10^ ^]^ while others merely describing the performance degradation or failure without providing a fundamental mechanistic explanation.^[^ ^9^, ^11^ ^]^ While this degradation is often vaguely attributed to an abrupt change in the thermal conditions and the disturbance of safe temperature gradients, no concrete evidence has supported this assumption. In practice, extensive engineering efforts have been devoted to mitigating thermal shock and improving system resilience upon temperature change, but these attempts have largely been unsuccessful, suggesting the existence of critical degradation mechanisms beyond our current understanding. Therefore, a fundamental scientific investigation is urgently required to uncover the root causes of this persistent challenge and develop truly effective solutions.

In this study, we discovered that air supply interruptions induce chemical degradation in cathode materials, which is unrelated to the presumed thermal variation mechanism and causes permanent and catastrophic cell damage. Through systematic model experiments, high‐resolution scanning transmission electron microscopy (STEM) analysis, and theoretical computation, we found that the abrupt cessation of air supply during operation rapidly decreases the oxygen partial pressure and creates conditions in which standard perovskite cathode materials, such as (La,Sr)CoO_3_ (LSC), chemically decompose within seconds. Notably, under these oxygen‐poor conditions, the Sr in the LSC plays a pivotal role in degradation, migrating and reacting with various nearby elements to induce harmful physical and chemical alterations. Leveraging our understanding of these mechanisms, we devised a practical solution by removing this critical element from the cathode. Experimental validation confirmed complete suppression of degradation during air‐supply interruption, underscoring the importance of our foundational insights in formulating an effective mitigation strategy.

## 2 Results and Discussion

### 2.1 Performance Assessment Under Air Supply Interruption

**Figure 1:**
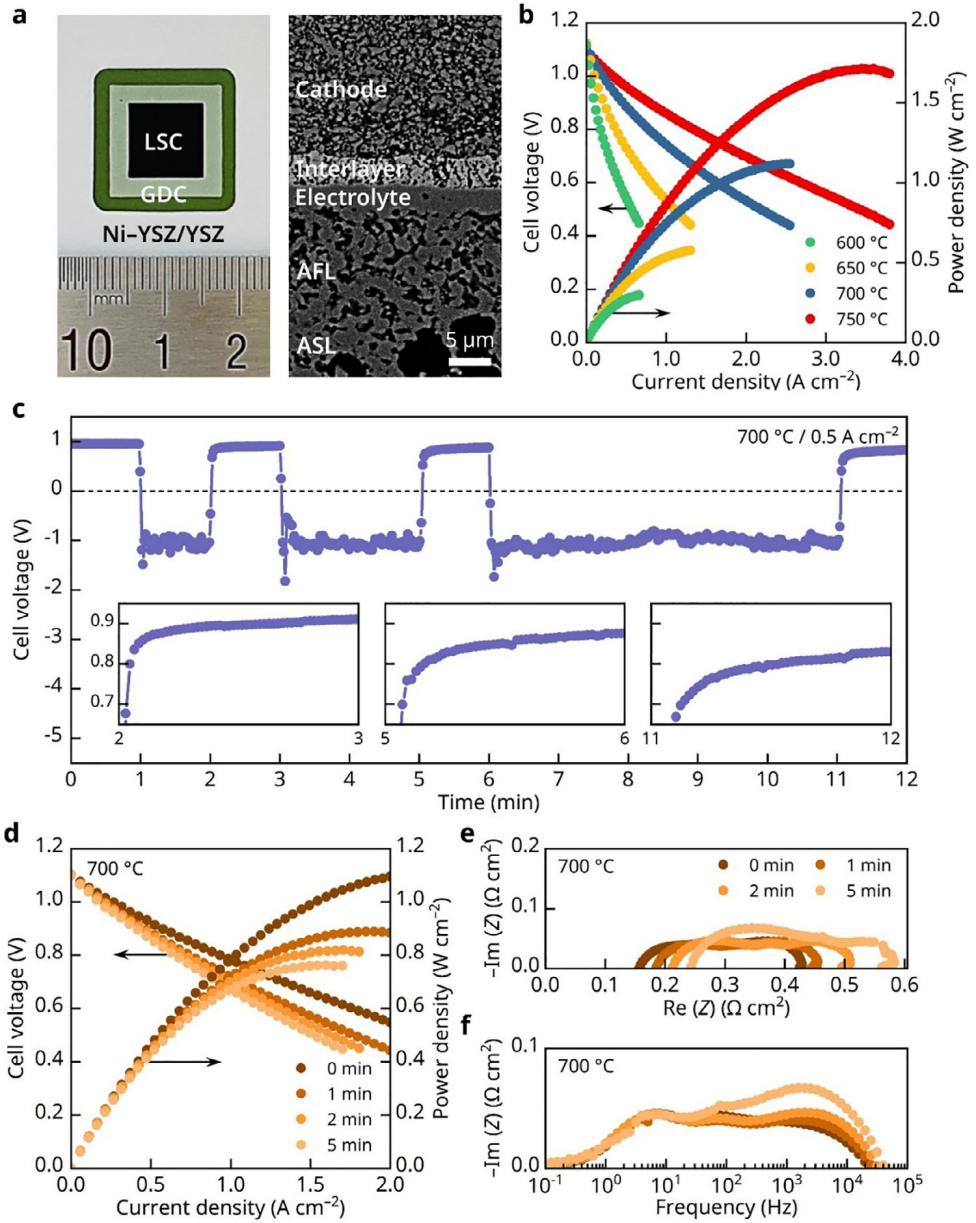
Air‐supply interruption tests for the SOFC. a) Photograph and cross‐sectional BSE image of the fabricated cell. b) Initial I–V–P curves measured at 600–750 °C. c) Cell voltage profiles during repeated air‐supply interruptions at 700 °C and 0.5 A cm^−2^ (insets: magnified views of voltage recovery behavior with increasing duration of air supply interruption). d) i–V–P curves measured at 700 °C with increasing air‐supply interruption duration. e) Nyquist plots of impedance spectra corresponding to (d). f) Bode plots of impedance spectra corresponding to (d).

The SOFC system consists of stacks and BOP components, including gas supply units, whose malfunction can result in a shortage of fuel or air during operation. Although a fuel supply shortage apparently causes Ni oxidation in the anode, the effects of air supply interruption on cathode performance are less obvious and have not been thoroughly studied. Contrary to the conventional view that degradation under air‐supply stoppage is primarily a thermal management issue—an approach that has thus far failed to deliver effective mitigation strategies—we propose that the intrinsic chemical stability of the cathode material constitutes a decisive factor. Traditional perovskite cathodes are known to be unstable under non‐ideal operating conditions, rendering them particularly vulnerable to degradation.^[^ ^12^ ^]^ For instance, the segregation of A‐site dopant cations in specific environments can increase the polarization resistance by up to two orders of magnitude.^[^ ^13^ ^]^ To assess the potential damage to the cathode caused by an air shortage, we experimentally simulated a scenario in which the air supply was interrupted during operation. Specifically, we fabricated an anode‐supported cell composed of a Ni–yttria‐stabilized zirconia (YSZ) anode support layer (ASL; ≈400 µm), a Ni–YSZ anode functional layer (AFL; ≈12 µm), a YSZ electrolyte (≈3.5 µm), a gadolinia‐doped ceria (GDC) interlayer (≈4.0 µm), and a lanthanum strontium cobaltite (LSC) cathode (≈30 µm). A photograph and the microstructure of the fabricated cell are shown in **Figure** **1**a. The electrochemical performance of the cell was evaluated using 3% humidified H_2_ as fuel and dry air as the oxidant. The current–voltage–power density (*I*–*V–P*) characteristics measured at 600–750 °C are shown in Figure 1b. The measured open‐circuit voltages (OCVs) were close to the theoretical values, confirming the leak‐tightness of the electrolyte and sealant. The fabricated cell exhibited maximum power densities of 0.30, 0.58, 1.1, and 1.7 W cm^−2^ at 600, 650, 700, and 750 °C, respectively, aligning well with reported values for standard SOFCs with high‐performance LSC cathodes.^[^ ^14^ ^]^ Electrochemical impedance spectra (EIS) (Figure S1, Supporting Information) further confirmed the normal electrochemical behavior of the tested cells.

Subsequently, we investigated the changes in the electrochemical characteristics caused by interruptions in the air supply at 700 °C. As shown in Figure 1c, we first operated the cell in galvanostatic mode at 0.5 A cm^−2^ under standard operating conditions at 700 °C, and the cell delivered a consistent voltage of ≈0.95 V, confirming stable performance with the normal uninterrupted air supply. When the air supply was halted while maintaining the current load, the cell voltage dropped abruptly into the negative range but recovered once air was reintroduced after 1 min. This procedure was repeated with extended interruption periods of 2 and 5 min, each time resulting in a sharp voltage drop during the stoppage, followed by recovery upon resumption. However, as clearly shown in the inset magnifications of Figure 1c, the recovered voltage progressively decreased after each interruption, indicating irreversible degradation caused by repeated air‐supply stoppages.

Our experiments, in which current was forced through the cell under halted air supply, causing the voltage to drop into the negative range, simulate the local severe conditions that can arise within a practical stack. In real stack operation, when the air supply is interrupted, the overall stack voltage and current will, in principle, gradually decline and eventually approach zero. However, during the transient period immediately following the air stoppage, the air distribution within the stack becomes highly non‐uniform, and certain regions can experience more rapid air depletion than others. Because the cells in the stack are connected in series, those locally starved cells rapidly lose their electromotive force but are momentarily forced to pass current driven by neighboring cells that remain operative. Under these conditions, the air‐depleted cells can exhibit negative voltages and are subjected to the most severe degradation, analogous to the highly resistive cells within stacks that have been reported to display negative voltages in the literature.^[^ ^15^ ^]^

Figure 1d presents the evolution of the *I–V–P* characteristics after successive air‐supply interruptions. The cell performance deteriorated progressively with each interruption, with the maximum power density declining from 1.1 to 0.76 W cm^−2^ after completion of the entire interruption tests. The changes in the EIS of the cell during the air stoppage are shown in Figure 1e,f. In the Nyquist plot in Figure 1e, the high‐frequency intercept and size of the impedance arc represent the ohmic and polarization resistances, respectively, both of which increased with the air stoppages. The impedance spectra of the SOFCs consist of multiple overlapping arcs, representing individual rate‐limiting processes with different characteristic frequencies. In the Bode plot of the imaginary part of the impedance in Figure 1f, the high‐frequency part increased significantly after the air stoppages, while the low‐frequency response was comparatively less affected. Typically, the high‐frequency impedance is associated with charge transfer and surface exchange reactions, whereas the low‐frequency impedance relates to gas‐phase diffusion.^[^ ^16^ ^]^ The notable increase in the high‐frequency impedance upon stopping the air supply suggests that the surface reaction degraded during operation without air, which was likely due to changes in the surface state of the cathode. In contrast, the low‐frequency impedance remained essentially unchanged by the air‐supply interruption, suggesting that the pore structure and gas diffusion processes were not significantly affected.

### 2.2 Structural and Chemical Analysis of Degradation Phenomena

**Figure 2:**
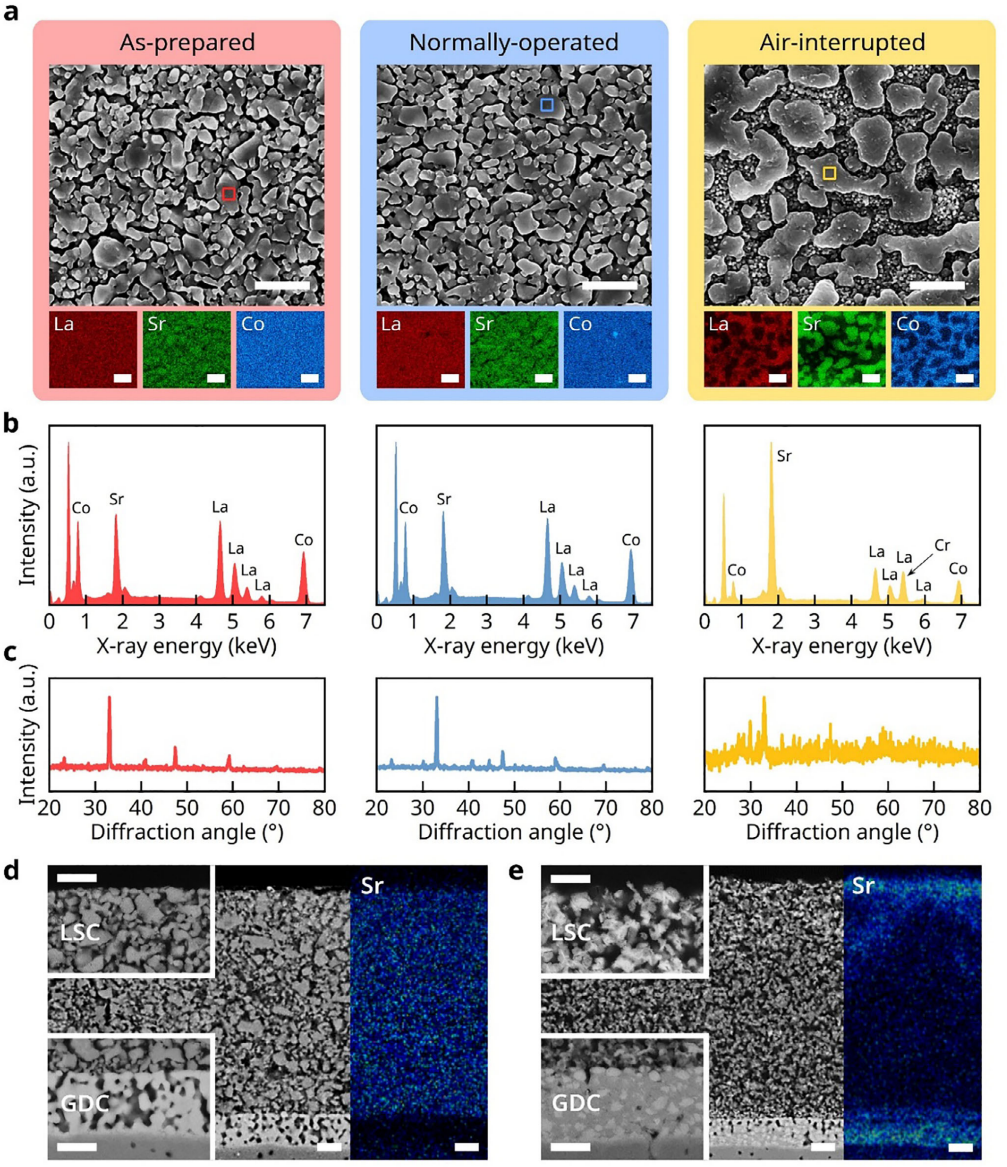
Postmortem analysis of the cathodes. a) SEM images with corresponding EDS elemental maps for La, Sr, and Co from the top surfaces of the as‐prepared cell (left), the cell operated under continuous air supply (middle), and the cell operated under interrupted air supply (right). b) EDS spectra acquired from the square‐marked regions in (a). c) XRD patterns collected from the same locations as in (a). d) Cross‐sectional BSE image and Sr elemental map of the cathode/interlayer region in the cell operated under continuous air supply. e) Cross‐sectional BSE image and Sr elemental map of the cathode/interlayer region in the cell operated under interrupted air supply. Scale bars in (a,d,e) represent 2 µm.

After the air‐interruption tests, the top surface of the cathode was examined by scanning electron microscopy (SEM) combined with energy‐dispersive X‐ray spectroscopy (EDS). For comparison, two additional identical cells were prepared: one analyzed in the as‐fabricated state and the other after operation under normal conditions without air interruption. The latter exhibited performance comparable to that of the cell subjected to air‐supply interruptions, as shown in Figure S2 (Supporting Information). The SEM images of the top surfaces of the cathodes are shown in **Figure** **2**a. After normal, uninterrupted operation, the cells did not significantly change with respect to the as‐fabricated cells. However, the cells subjected to air interruptions were noticeably altered, with large, abnormal fragments formed on the cathode surface. The highly magnified image in Figure S3 (Supporting Information) reveals that the particles beneath these large fragments were considerably finer than the initial LSC particles. In the EDS analysis presented in Figure 2b and Table S1 (Supporting Information) both the as‐fabricated and normally operated cathodes exhibit compositions consistent with the nominal stoichiometry of LSC (La_0.6_Sr_0.4_CoO_3‐δ_), indicating that this phase remained stable under uninterrupted operation. In contrast, the air‐interrupted cathode shows pronounced Sr and Cr enrichment in regions containing large abnormal fragments, accompanied by a corresponding depletion of La and Co. The EDS elemental maps in Figure S4 (Supporting Information) show that the fine particles located beneath the large fragments are enriched in La and Co. During cell testing, we used ferritic stainless‐steel interconnects, which emit Cr vapor and contaminate the cathode. Evaporated Cr species are known to react preferentially with the SrO segregated from conventional LSC cathodes, forming Sr–Cr–O phases.^[^ ^17^ ^]^ We hypothesized that the air stoppage during operation caused the formation of Sr‐rich phases and promoted interactions with Cr vapor, leading to the appearance of the abovementioned large fragments on the top surface. The formation of Sr–Cr–O compounds on the electrode surface was further confirmed by Raman spectroscopy (Figure S5, Supporting Information), which revealed a distinct SrCrO_4_ peak at ≈860 cm^−1^.^[^ ^18^ ^]^ The XRD analysis, shown in Figure 2c, confirmed that the perovskite LSC structure did not significantly change during normal operation. However, the XRD pattern of the cell subjected to air interruption displayed indistinct peaks with considerable noise, indicating the breakdown of the perovskite crystal structure. The XRD analysis could not identify the crystal structure of the reaction product on the top surface.

Next, we analyzed and compared cross‐sections of the cells that were both operated under normal conditions except for the continuous and interrupted air supplies. As shown in Figure 2d, the cell tested under uninterrupted operating conditions displayed typical microstructural and chemical characteristics. This cell consisted of a dense YSZ electrolyte, a porous GDC interlayer, and a porous LSC cathode, with the constituent elements of each layer mostly uniformly distributed within their respective layers. In contrast, the cells that experienced air interruption exhibited significant changes in both the microstructure and elemental distribution (Figure 2e). The most notable feature was the nonuniform distribution of Sr, with strong Sr signals detected at the top and bottom of the cathode—specifically on the surface of the cathode and near the GDC interlayer—whereas less Sr appeared in the middle of the cathode. A significant amount of Sr was also detected within the GDC interlayer. This nonuniform Sr distribution primarily resulted from Sr segregation toward the surfaces of the LSC particles, triggered by the air stoppage. Sr segregation is well known to be thermodynamically favorable^[^ ^19^ ^]^ and occurs more readily under low oxygen partial pressures.^[^ ^20^ ^]^ Prolonged air interruption increases the local temperature of the power‐generating cell and exposes the LSC cathode to a low‐oxygen environment, which encourages Sr segregation and subsequent Sr migration towards the surface and interface.^[^ ^21^ ^]^ In the backscattered electron (BSE) image of this cell (Figure 2e), the LSC particles in the middle section became noticeably finer with nonuniform contrast, which indicates that these fine particles consisted of several distinct parts with varying chemical compositions (see Figure S6, Supporting Information for magnified images). Furthermore, the pores in the initially porous GDC layer were nearly filled with unidentified materials that had a darker contrast than the original GDC phase. Overall, the postmortem analysis in Figure 2 demonstrates that the physical and chemical features of the entire LSC cathode and the GDC interlayer changed significantly owing to the series of air interruptions.

**Figure 3:**
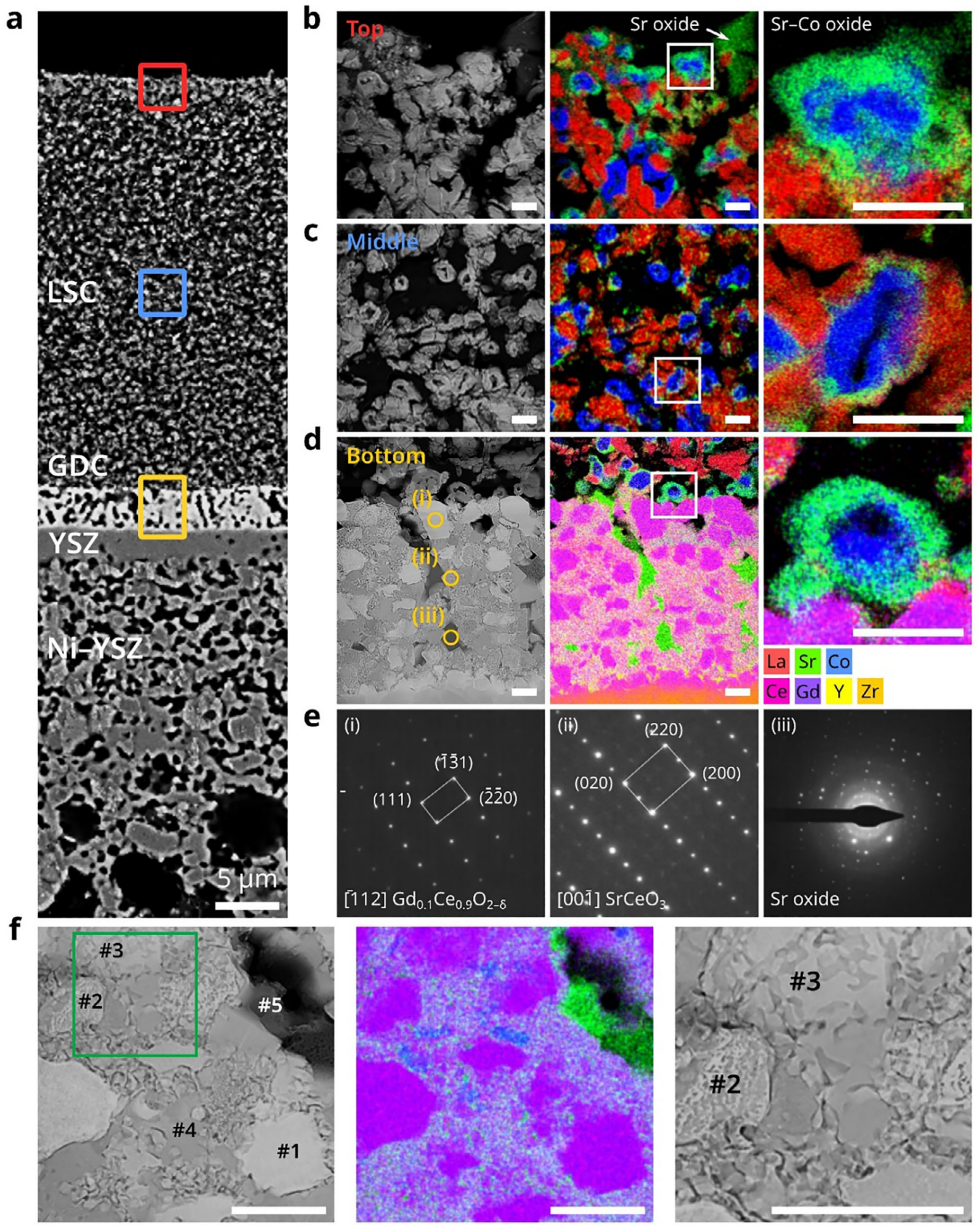
STEM–EDS analysis of structural degradation at different depth within the cathode of a cell subjected to air‐supply interruptions. a) Cross‐sectional BSE‐SEM image showing the regions selected for STEM analysis. HAADF–STEM image and corresponding EDS elemental maps of the b) top (red box in (a)), c) middle (blue box in (a)), and d) bottom (yellow box in (a)) regions with magnified views of a Co‐rich oxide with a hollow center on the right. e) SAED patterns from the bottom region in (d), identifying (i) Gd_0.1_Ce_0.9_O_2‐δ_, (ii) SrCeO_3_, and (iii) Sr oxide. f) HAADF–STEM image (left), corresponding EDS elemental map (middle), and magnified view of the green‐boxed region in the left image (right). Scale bars in (b–d) and (f) represent 500 nm.

To acquire more detailed information about the degradation phenomena, STEM–EDS analysis was carried out on the cell subjected to air‐supply interruption. The sample was taken from the air‐inlet region, where degradation was most pronounced, as indicated by the yellow square in Figure S7 (Supporting Information). From this region, TEM specimens were prepared at three positions along the vertical direction of the cathode—top, middle, and bottom—as marked by the red, blue, and yellow squares in the SEM image in **Figure** **3**a. This analysis revealed that the LSC cathode was almost completely decomposed, and its constituent elements (La, Sr, and Co) were unevenly distributed throughout the cathode. Furthermore, STEM–EDS analysis of the top, middle, and bottom sections shown in Figure 3b–d, respectively, revealed distinct degradation characteristics. The middle (Figure 3c) exhibited chemical phase separation, which resulted in La, Sr, and Co existing mostly as distinct entities. In addition, various decomposition products were identified, offering insights into the degradation mechanism. Notably, Co‐rich oxide was frequently observed in the form of donut‐shaped particles with a hollow core (Figure 3b–d). These oxides were enveloped by a Sr‐rich surface layer and accompanied by nearby La‐rich oxide particles, suggesting that degradation occurred through the outward diffusion of A‐site elements, predominantly driven by Sr. This left behind Co‐rich oxide particles with hollow cores that retained the hexagonal crystal structure, with lattice parameters of *a* = 0.58 nm and *c* = 0.95 nm and grain sizes ranging from 1 to 200 nm. The segregation of A‐site cations to the surface, particularly Sr, has been reported as a major degradation pathway in perovskite oxides.^[^ ^22^ ^]^ TEM analysis in Figure 3 reveals that this outward migration of Sr was markedly accelerated under air interruption conditions, resulting in complete chemical disintegration. A key driving force for this Sr segregation is considered to be the electrostatic attractive force exerted by surface oxygen vacancies. In LSC, Sr^2+^ substitutes for La^2+^, introducing a net negative charge ($Sr_{La}^{\prime }$) that makes Sr susceptible to attraction by the positively charged oxygen vacancies ($V_{O}^{••}$).^[^ ^23^ ^]^ During air interruption, the rapid depletion of oxygen instantaneously forms a large number of surface oxygen vacancies, creating a strong attractive electrostatic force that pulls Sr toward the surface. The migration of Sr toward the electrode surface is further corroborated by our density functional theory (DFT) calculations (Figure S8, Supporting Information). Specifically, the surface segregation energy (*E_seg_*) of pristine LSC was calculated to be –0.89 eV, which further decreases to –1.29 eV in the presence of a surface oxygen vacancy. The lower *E_seg_* implies that Sr segregation becomes more thermodynamically favorable, indicating that the formation of surface oxygen vacancies upon air supply interruption promotes Sr migration toward the LSC surface. This process results in rapid chemical decomposition, with a distinct morphology wherein Sr accumulates around the periphery of Co. Moreover, the increased oxygen vacancy concentration substantially lowers the activation energy for Sr diffusion, thereby promoting its migration toward the surface and progressively accelerating the decomposition of LSC over time.^[^ ^24^ ^]^ The top section (Figure 3b) had a significantly high Sr concentration, where Sr existed in two forms: Sr–Co oxide encasing the hollow Co oxide structures and micrometer‐scale Sr oxide particles in the uppermost region (highlighted by the white arrow in Figure 3b). Owing to its high Sr content, the Sr–Co oxide layers surrounding the hollow Co oxides were considerably thicker than those observed in the middle section. At the bottom of the cathode (Figure 3d), just above the interlayer, the decomposition of LSC produced Co‐rich oxides surrounded by Sr–Co oxides, similar to the top region.

Meanwhile, the initially porous GDC interlayer beneath the LSC cathode transformed into a dense layer composed of four components: the original GDC, Sr‐containing GDC, SrCeO_3_, and Sr oxide. STEM–EDS analysis revealed that the brightest particles in the high‐angle annular dark‐field (HAADF)–STEM image (region (i) in Figure 3d and region #1 in Figure 3f) were similar to the original GDC, although their proportion significantly decreased compared to the initial state before air interruption. Instead, the gray regions became dominant, containing Sr along with Ce and Gd. This implies that Sr reacted with GDC to form a new phase, transforming the porous structure into a dense layer. This process likely involved a transient, liquid‐like phase generated at the operating temperature in the absence of air, which spread through capillary action. These gray regions exhibited a complex microstructure (Figure 3). Notably, the EDS results of area #4 indicate a 1:1 Sr‐to‐Ce ratio (**Table** **1**), which was confirmed to be the SrCeO_3_ phase by the selected‐area electron diffraction (SAED) pattern (region (ii) in Figure 3e). However, the other particles (#2 and #3 in Figure 3f), identified as GDC in the EDS maps, displayed irregular and variable compositions between those of #1 and #4 (Table 1). Additionally, particle #3 in Figure 3f exhibited an interconnected structure characteristic of spinodal decomposition in liquid phases between elements with a positive heat of mixing, as evident in the magnified view on the right of Figure 3f.^[^ ^25^ ^]^ Because Sr and Gd must be thermodynamically immiscible, similar to the reported immiscibility of Sr and Nd,^[^ ^26^ ^]^ the GDC particles mixed with Sr underwent phase separation, and their structure and composition varied with the Sr content. These findings suggest that Sr and Gd entered a liquid state during the air‐off operation. The dark regions in the HAADF–STEM image correspond to Sr oxide, likely originating from the Sr that migrated from the LSC, which remained unreacted with the GDC and was embedded within the interlayer. Overall, the high‐resolution STEM analysis clearly indicates that Sr played a pivotal role in the chemical decomposition of LSC, driving various subsequent degradation phenomena.

**Table 1:** Atomic compositions (at %) at points #1 – #5 within the GDC interlayer regions shown in Figure 3 f, determined by STEM–EDS analysis. The relatively low quantitative oxygen values are attributed to oxygen absorption effects associated with sample thickness during STEM–EDS measurements.

Spot	O	Sr	Ce	Gd	Expected phases
#1	47.8	0.14	43.6	8.42	Gd_0.1_Ce_0.9_O_2‐δ_ (GDC)
#2	49.6	1.59	41.1	7.72	Sr‐containing GDC
#3	46.4	6.29	40.7	6.66	Sr‐containing GDC
#4	40.1	29.6	26.0	4.32	SrCeO_3_
#5	41.9	57.1	–	1.03	Sr oxide

### 2.3 Dynamic Multiphysics Modeling of Air Supply Interruptions

**Figure 4:**
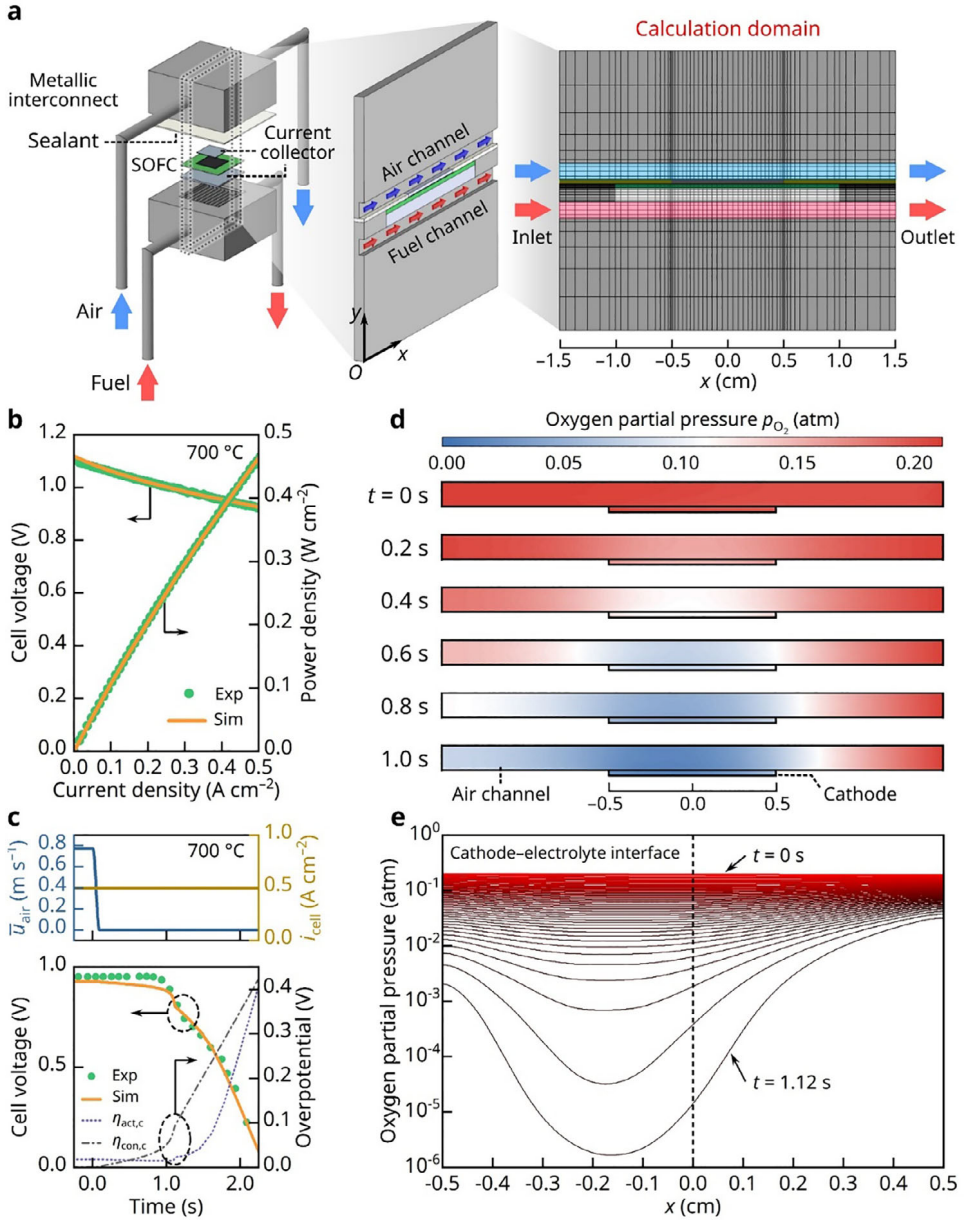
Dynamic simulation of SOFC behavior during air‐supply interruption. a) Schematic of the developed 2D dynamic multiphysics model and computational domain. b) Model validation by comparison of simulated and experimental *I–V–P* curves under continuous air supply. c) Time‐dependent profiles of air inlet velocity, applied current density, and corresponding experimental/simulated cell voltages, along with calculated activation and concentration overpotentials in the cathode. d) Temporal evolution of the oxygen partial pressure distribution across the cathode and air channel. e) Oxygen partial pressure profiles at the cathode–electrolyte interface over time.

To understand the environmental changes that drive cathode degradation upon air stoppage, we developed a 2D dynamic multiphysics model, as detailed in the Methods section. This model incorporates key aspects of cell testing, including the configuration, components, and experimental geometry (**Figure** **4**a). The developed model was validated by the good agreement between the experimental and simulated *i*–*V*–*P* characteristics at 700 °C (Figure 4b). We then simulated the transient response of the power‐generating cell to air interruption. As shown in Figure 4c, the simulated voltage evolution over the 2‐second window closely matches the experimental results, accurately capturing the dynamic behavior of the cell. Following air interruption, the rapid drop in the oxygen partial pressure progressively increases both the activation and concentration overpotentials in the cathode, which indicate decreases in the surface reaction kinetics and gas transport rate, respectively, thereby decreasing the cell voltage.

To analyze this transient behavior in detail, we investigated the changes in the oxygen distribution within the cathode and air channels over time. Figure 4d illustrates that after the air supply is cut off, the oxygen distribution gradually loses the uniformity observed at the initial stage (*t* = 0 s). In particular, near the air inlet, the oxygen partial pressure rapidly declines to extremely low levels where the LSC cathode becomes susceptible to chemical decomposition. Specifically, at the cathode–electrolyte interface, the oxygen partial pressure drops as low as 10^−6^ atm within seconds (Figure 4e). As stated in the previous section, severe oxygen depletion induces abundant positively charged oxygen vacancies ($V_{O}^{••}$) at the surface of LSC that drive outward diffusion of negatively charged Sr ions ($Sr_{La}^{\prime }$), causing continuous Sr segregation and eventual electrode decomposition. Additionally, the limited external oxygen supply cannot sustain the ionic current, forcing LSC to consume its lattice oxygen in a process analogous to electrochemical oxygen pumping in nonstoichiometric oxides.^[^ ^27^ ^]^ This, in turn, further accelerates Sr segregation and the chemical decomposition of the LSC cathode, representing an additional mechanism for Sr segregation beyond the elastic strain and electrostatic effects.^[^ ^23a^ ^]^ On the outer top surface of the cathode, segregated Sr reacts with Cr vapor to form SrCrO_4_, as confirmed by SEM–EDS (Figure 2) and Raman (Figure S5, Supporting Information) analyses. Thermodynamic assessments indicate that SrCrO_4_ formation is favored under oxidizing conditions above ≈10^−6^ atm *pO_2_*, while more reduced Sr–Cr oxides such as SrCrO_3_, Sr_3_Cr_2_O_8_, and SrCr_2_O_4_ are stable at lower *pO_2_*.^[^ ^28^ ^]^ As shown in Figure S9 (Supporting Information), multiphysics modeling reveals that the outer cathode surface remains less reducing (≈3.7×10^−5^ atm) than the cathode–electrolyte interface (≈1.6×10^−6^ atm, Figure 4e), making SrCrO_4_ formation thermodynamically feasible on the top surface. Nonetheless, we cannot fully exclude the transient formation of reduced Sr–Cr oxides during air interruption, followed by their oxidation to SrCrO_4_ upon reintroduction of air—representing an alternative pathway for SrCrO_4_ formation. Notably, when oxygen inflow at the inlet is completely halted, the oxygen partial pressure decreases more slowly near the outlet than at the inlet owing to backflow of air from the outlet. This phenomenon explains the more pronounced degradation typically observed near the air‐inlet region in commercial‐scale large cells, as discussed later.

To further analyze the dynamic evolution of the oxygen environment and the associated changes in material properties, we performed time‐domain fitting of the voltage recovery behavior following air supply resumption using a first‐order exponential model:

(1) $$V_{cell}=A\left(1-\exp\left(-\frac{t}{\tau }\right)\right)+B$$

where 𝑉_cell_ is the cell voltage, 𝑡 is time, τ is the time constant (i.e., relaxation time), and A and B are fitting parameters. As shown in Figure S10 (Supporting Information), the model successfully simulated the relaxation behavior, yielding time constants of 0.634, 0.704, and 0.777 s for air interruption durations of 1, 2, and 5 min, respectively. These values (≈0.6–0.8 s) are in good agreement with the characteristic time scale predicted by the multiphysics simulation in Figure 4e, which indicates that the oxygen partial pressure at the cathode–electrolyte interface falls below the critical decomposition threshold within ≈1 second of air interruption. This correlation provides strong evidence that rapid oxygen depletion and subsequent lattice oxygen dynamics are key drivers of cathode degradation. Furthermore, the observed increase in relaxation time with longer air interruption durations suggests that more severe lattice oxygen depletion occurs during extended oxygen deprivation, thereby requiring a longer time for re‐equilibration upon air reintroduction.

It should be noted that this multiphysics model simulates the early‐stage evolution of environmental conditions that trigger cathode degradation—namely, the time window from the onset of air supply interruption to the point where the oxygen partial pressure drops below the critical threshold for LSC decomposition. Simulating the degradation that follows LSC decomposition is significantly more complex because it requires detailed knowledge of the properties of the decomposition products and how these evolve over time, which remains difficult to quantify experimentally or computationally. Performance degradation resulting from phase decomposition is primarily governed by two factors: changes in material properties and alterations in microstructure. While modeling the effects of evolving material properties remains highly challenging due to the lack of reliable data for decomposition products, the impact of microstructural changes can be reasonably estimated. As shown in Figure S11 (Supporting Information), we performed simulations to evaluate the impact of porosity and permeability variations on cell performance. Variations of up to ±20% in these parameters led to minimal voltage changes—less than 0.22%—indicating that performance degradation during LSC decomposition is primarily driven by the loss of electronic and catalytic functionality due to phase decomposition, rather than by alterations in gas transport properties.

### 2.4 Commercial‐Scale Cell Evaluation Under Air Supply Interruption

**Figure 5:**
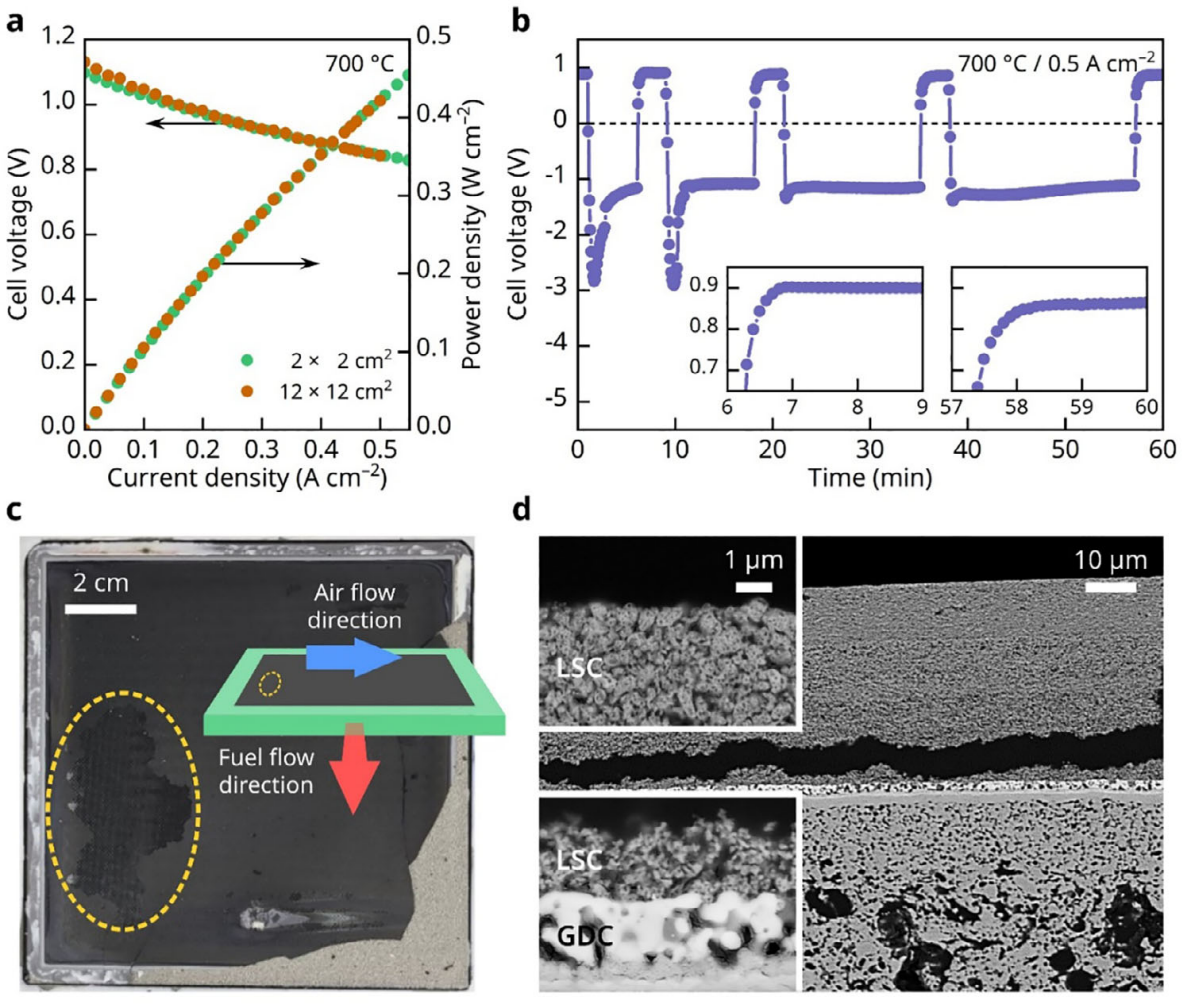
Air‐supply interruption tests on large‐area SOFCs. a) Initial i–V curves and corresponding power density profiles of a button cell (2 × 2 cm^2^) and a practical‐scale large cell (12 × 12 cm^2^) measured at 700 °C. b) Cell voltage response under a constant current density of 0.5 A cm^−2^ during repeated air‐supply interruptions (insets: magnified views of voltage recovery after interruptions). c) Photograph of the practical‐scale SOFC, with the yellow oval indicating the air inlet region from which the sample was taken for cross‐sectional analysis. d) Cross‐sectional BSE images of the sampled region, showing the LSC cathode layer and underlying GDC interlayer.

An additional air interruption experiment was carried out on a commercial‐scale large cell with an active area of 100 cm^2^ to investigate the lateral distribution of degradation. As shown in **Figure** **5**a, the large cell initially performed comparably to a button cell, confirming the effectiveness of our testing method and demonstrating no performance loss due to the increased cell size.^[^ ^29^ ^]^ We then performed the air interruption test using the same protocol as that for the lab‐made button cell. As shown in Figure 5b, the cell performance progressively degraded with the increasing air interruption time, and the recovery time also extended with longer interruptions, exhibiting a trend similar to that observed in the button cell. A key distinction, however, is that both the degradation and recovery rates—reflected in the cell voltage change—were slower in the large cell. Postmortem analysis revealed that the apparently slower degradation rate in the large cell did not indicate reduced overall degradation. Rather, it reflects the non‐uniform spatial distribution of oxygen depletion, which causes highly localized degradation—resulting in a slower average voltage decline but more severe damage in specific regions. As highlighted by the yellow oval in Figure 5c, the cathode severely delaminated near the air inlet region, reflecting the spatial variation in the oxygen partial pressure. In our testing configuration, when the airflow was blocked at the inlet, the outlet remained open to the ambient air, allowing oxygen to backflow from outside. This suggests that oxygen was more likely to be depleted near the inlet, while some oxygen may still have reached the outlet region through this backflow. Consequently, the oxygen partial pressure decreased more rapidly at the inlet, more severely damaging this region, as predicted by the simulation (Figure 4d,e). The BSE image in Figure 5d shows the secondary reaction products formed by the interaction between Sr and GDC within the GDC interlayer, similar to the findings from the button cell (Figure 2e). Additionally, the cathode–electrolyte interface was severely fractured, indicating that the chemical degradation of the cathode and interlayer compromised the mechanical integrity of the cell, ultimately leading to catastrophic structural failure. The interfacial fracture observed in the large‐sized cell closely corresponds to the degradation phenomena identified in the button cell experiments. TEM analysis of the button cell in Figure 3 revealed that the LSC cathode undergoes phase decomposition into discrete La‐, Sr‐, and Co‐based oxides. Simultaneously, the GDC interlayer reacts with Sr that has migrated from the cathode, resulting in disintegration into nanocrystalline secondary phases. This chemical degradation significantly compromises the mechanical integrity of the electrode structure. The collapse of the original perovskite framework and the formation of mechanically weaker phases introduce local stress concentrations, which, under electrochemical and thermal loading, lead to crack initiation and ultimately interfacial mechanical failure.

### 2.5 Development of a Sr‐Free Electrode as a Mitigation Strategy

Our investigation identified Sr as the key driver of the severe chemical degradation observed in LSC cathodes. Under reduced pO2 conditions during air‐supply interruption, Sr strongly promotes decomposition of the perovskite structure and accelerates detrimental secondary reactions. Therefore, eliminating Sr from the cathode composition is expected to substantially enhance stability and durability under such conditions. However, most Sr‐free cathode materials have failed to match the performance of Sr‐containing counterparts because the partial substitution of La^3+^ with lower‐valence Sr^2+^ is critical for achieving essential electrode properties, such as catalytic activity and ionic and electronic conductivities.^[^ ^30^ ^]^ Because it is highly challenging for a single Sr‐free material to simultaneously exhibit all the required properties, we adopted a composite design strategy, combining La_2_NiO_4+δ_ (LNO) and LaNi_0.6_Co_0.4_O_3–δ_ (LNC) to decouple and optimize the electronic and ionic transport properties. LNO, the first member of the Ruddlesden–Popper series, features alternating LaO rock salt and LaNiO_3_ perovskite layers.^[^ ^31^ ^]^ The open structure of the rock salt layer facilitates oxygen ion transport,^[^ ^32^ ^]^ although LNO suffers from relatively low electronic conductivity. In contrast, LNC exhibits high electronic conductivity, up to ≈1200 S cm^−1^ at 800 °C,^[^ ^33^ ^]^ enabled by the narrow itinerant conduction band formed by the Ni arrays.^[^ ^34^ ^]^ By compositing LNO and LNC, we synergistically combined their high ionic and electronic conductivities.

**Figure 6:**
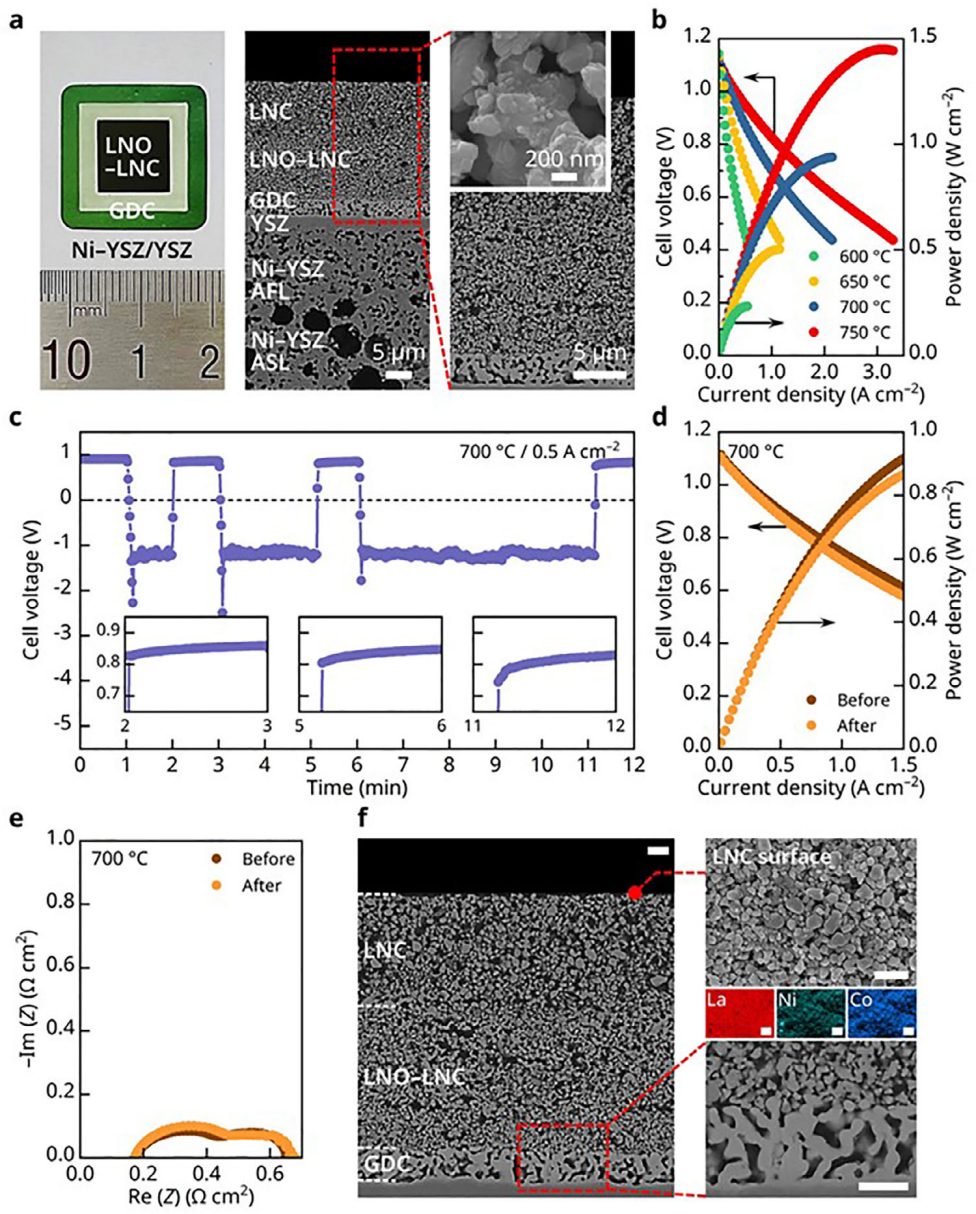
Air‐supply interruption tests on the Sr‐free cathode. a) Photograph and cross‐sectional BSE images of the cell incorporating an Sr‐free cathode. b) Initial I–V–P curves and corresponding power density profiles measured at 600–750 °C. c) Cell voltage response under a constant current density of 0.5 A cm^−2^ during repeated air‐supply interruptions (insets: magnified views of voltage recovery after interruptions). d) I–V–P curves and e) Nyquist plots of impedance spectra recorded before and after air‐supply interruption at 700 °C. f) Cross‐sectional SEM images after air‐supply interruption, showing the LNC cathode surface (top) and the cathode–interlayer interface (bottom), with corresponding EDS elemental maps for La, Ni, and Co. Scale bars in (f) represent 2 µm.

Moreover, to further enhance the surface exchange kinetics, we introduced LNC nanocatalysts using our advanced infiltration technique.^[^ ^35^ ^]^ In this process, a chemical solution is injected into the porous electrode structure, and the desired nanoscale catalyst particles are formed in situ via subsequent thermal treatment. Unlike conventional methods that require high calcination temperatures (>900 °C) to achieve phase purity, our technique enables the formation of complex, multi‐element oxides with exceptional phase purity at significantly lower temperatures (≈650 °C).^[^ ^36^ ^]^ This results in ultrafine, uniformly distributed nanoparticles with negligible thermal aggregation. The key to this technique lies in the use of appropriate complexing agents that ensure the homogeneous precipitation of multiple elements. Using urea and glycine as complexing agents, we successfully synthesized phase‐pure LNC nanocatalysts, as confirmed by the XRD analysis in Figure S12 (Supporting Information). A photograph and SEM images of the fabricated cell are shown in **Figure** **6**a. This cell incorporated a bilayer Sr‐free cathode consisting of a ≈10 µm thick LNC current collecting layer deposited over a ≈10 µm thick LNO–LNC functional layer. The infiltrated LNC nanocatalysts were homogeneously distributed along the inner surfaces of the porous structure, as depicted in the inset of Figure 6a and in Figure S13 (Supporting Information). All other cell components were identical to those used in previous tests. This cell with Sr‐free cathode exhibited excellent electrochemical performance, achieving a peak power density of ≈1 W cm^−2^ at 700 °C (Figure 6b), comparable to that of a reference cell with an LSC cathode. We also subjected this cell to air interruption tests identical to those previously conducted with LSC. After up to 5 min of air supply interruption, the cell voltage fully recovered upon reintroduction of air with minimal degradation (Figure 6c). Neither the *i–V–P* characteristics (Figure 6d) nor the EIS results (Figure 6e) showed any changes, demonstrating a strong ability to withstand an oxygen shortage. The SEM analysis (Figure 6f) revealed no structural abnormalities or surface fragmentations following the interruption tests. Additional SEM/EDS analyses in Figures S14 and S15 (Supporting Information) confirmed that the surface structure and composition remained unchanged.

Finally, to assess the durability of the Sr‐free cathode under extreme conditions, we performed extended air‐supply interruption tests by halting the air supply for 30 min while maintaining a constant current. As shown in Figure S16 (Supporting Information), the cell fully recovered its performance after the 30 min interruption, with no observable degradation, and continued stable operation thereafter. As noted earlier, this test—forcing current flow in the absence of air supply—simulates the worst‐case scenario for a specific cell in a stack experiencing the most rapid oxygen depletion, in which the current is driven by neighboring cells with no oxygen supply, leading to a temporary negative cell voltage. In a real system operation, such a condition would occur only momentarily before the current ceases or the air supply is restored. The ability of our Sr‐free cathode to withstand 30 min of continuous current flow without air supply highlights its potential to effectively address air‐supply interruption issues in practical applications.

## 3 Conclusion

With the rapid commercialization of SOFCs, various practical issues have arisen in real‐world operation. Until now, air supply problems have received little attention, although they have been frequently found to impact the reliability of SOFC systems during operation. This study revealed that even briefly interrupting the air supply can severely damage the cathode. The passage of continuous electric current under insufficient air supply conditions significantly decreases the oxygen partial pressure, which chemically decomposes standard perovskite cathode materials. Sr was identified as the primary cause of demixing and undesirable reactions, which suggested that using a Sr‐free cathode material would be an effective mitigation strategy to address air supply interruption issues. While the effectiveness of this approach has been validated, further research is necessary to enable the practical implementation of Sr‐free cathodes in full‐scale cells and stacks.

These findings clearly depart from the prevailing but vague assumption that thermal fluctuations are the main cause of degradation following air supply interruptions. Instead, they reveal a fundamentally different degradation mechanism that provides a novel and effective pathway for addressing this critical issue. Our study underscores the need for a new direction in fundamental research—one that prioritizes understanding and solving practical challenges arising during real‐world operation.

## 4 Experimental Section

### Experimental Procedures

Air interruption tests were conducted using a 2 × 2 cm^2^ anode‐supported SOFCs. For cell fabrication, 54 wt.% NiO (FUJIFILM Wako Pure Chemical Corp., Japan), 36 wt.% (Y_2_O_3_)_0.08_(ZrO_2_)_0.92_ (YSZ) (TZ‐8Y, Tosoh Corp., Japan), and 10 wt.% poly(methyl methacrylate) (SUNPMMA‐S50, Sunjin Beauty Science Co., Ltd., Korea) powders were mixed in ethanol with Triton X‐100 (Daejung Chemicals & Metals Co., Ltd., Korea) as a dispersant, polyvinyl butyral (Butvar B‐76, Eastman Chemical Company, USA) as a binder, and polyethylene glycol 400 and glycerin (Daejung Chemicals & Metals Co., Ltd.) as plasticizers. This mixture was then ball‐milled for 48 h. The anode support was prepared by tape‐casting the slurry onto a glass substrate. After drying, the tape was cut and pre‐sintered at 1000 °C for 2 h. Next, a fine‐grade NiO (Sumitomo Metal Mining Co., Ltd., Japan)–YSZ (NiO/YSZ = 66:34 wt.%) anode functional layer and a YSZ electrolyte were sequentially applied by spin coating, followed by co‐sintering at 1330 °C for 5 h. Finally, a Gd_0.1_Ce_0.9_O_1.95_ (GDC) interlayer (GDC LSA, Solvay S.A., Belgium) and a La_0.6_Sr_0.4_CoO_3‐δ_ (LSC) cathode (LSC64‐F, Kceracell Co., Ltd., Korea) were formed with lab‐made screen‐printable inks, which were sequentially sintered at 1250 and 950 °C for 2 h at each temperature.

For electrochemical testing, the fabricated cells were positioned between metallic testing fixtures made of Inconel 600 (Special Metals Corp., USA) and sealed using a glass–ceramic sealant. Ni foam and Pt mesh served as current collectors for the anode and cathode, respectively. The cells were tested under 3% humidified H_2_ as fuel and air as the oxidant, with the electrochemical performance evaluated at 600–750 °C. Measurements were conducted using a Solartron 1260A frequency response analyzer and a Solartron 1287A potentiostat/galvanostat (Solartron Analytical, UK). For the air interruption tests, the air supply was abruptly halted for a specified duration and then resumed while maintaining a constant current density of 0.5 A cm^−2^. The cell voltage was monitored throughout the process at 700 °C, and *i–V* characteristics and EIS were recorded after each cycle. A practical‐scale 12 × 12 cm^2^ anode‐supported SOFC (ASC‐400B, Elcogen AS, Estonia) was also tested following the same air‐interruption procedure.

For the cell with the Sr‐free cathode, LNO powder was synthesized using the glycine–nitrate combustion method. Specifically, stoichiometric amounts of La(NO_3_)_3_·6H_2_O (Daejung Chemicals & Metals Co., Ltd.) and Ni(NO_3_)_2_·6H_2_O (Daejung Chemicals & Metals Co., Ltd.) were dissolved in purified water, and glycine (Daejung Chemicals & Metals Co., Ltd.) was added as a combustion fuel at a glycine‐to‐nitrate ratio of 0.55:1. The mixture was placed in a stainless‐steel beaker and subjected to autoignition at 550 °C using a hot plate. The obtained ash was ball‐milled in ethanol for 24 h, dried, sieved with a 100 µm mesh, and then calcined at 950 °C for 2 h. The LNC powder was purchased from Nexceris LLC, USA, and used as received. Powder XRD analysis was performed using Cu Kα radiation using a D8 Advance diffractometer (Bruker Corp., USA). To fabricate the cell, the same anode‐supported bilayer with the NiO–YSZ/YSZ/GDC structure described earlier was used as the substrate, and an LNO–LNC cathode and an LNC current collecting layer (Nexceris LLC, USA) were sequentially applied by screen printing, followed by sintering at 950 °C for 2 h. For infiltration, the precursor solution of LNC was prepared by mixing La(NO_3_)_3_·6H_2_O, Ni(OCOCH_3_)_2_·4H_2_O and Co(NO_3_)_2_·6H_2_O (Sigma Aldrich) in distilled water and ethanol at a concentration of 0.5 mol L^−1^. Glycine was then dissolved at a glycine‐to‐cation ratio of 1:1, followed by the addition of urea (Sigma–Aldrich) at a urea‐to‐cation ratio of 10:1. The precursor solution was infiltrated into the air electrode, and the cell was heat‐treated at 650 °C for 2 h.

After the air interruption tests, the cells were analyzed using a D/MAX‐2500 X‐ray diffractometer (Rigaku Corp., Japan) and field‐emission (FE)–SEM (Regulus8230, Hitachi High‐Tech Corp., Japan) with EDS (Oxford Instruments plc, UK). For the cross‐sectional analysis, the cells were impregnated with epoxy resin (EpoFix, Struers, Denmark) and then cut, polished, and coated with Pt. Nanoscale STEM analysis was performed using a Talos F200X (Thermo Fisher Scientific Inc., USA) equipped with EDS (Super‐X, Bruker Corp.). For phase identification, SAED patterns were obtained. TEM lamella samples were prepared using a focused ion beam (FIB) (Ethos NX5000, Hitachi High‐Tech Corp.).

### Computational Details

A 2D multiphysics numerical model was developed to simulate the transient response of SOFCs during air supply interruption, building upon prior 3D steady‐state^[^ ^37^ ^]^ and transient^[^ ^38^ ^]^ models and a 1D microstructurally resolved model.^[^ ^36^ ^]^ The 2D model incorporated the geometric dimensions (Table S2, Supporting Information), governing equations (**Table** **2**), constitutive relations (**Table** **3**), material properties (Table S3, Supporting Information), and microstructural parameters (Table S4, Supporting Information) of the experimental cells. These models are described in detail in the literature.^[^ ^37^, ^38^, ^39^ ^]^

**Table 2:** Governing equations.

	Governing equation	Domain
Electron	$\nabla \cdot \left(-\sigma_{e^{-}}^{\mathrm{eff}}\nabla \phi_{e^{-}}\right)=-S^{\mathrm{eff}}i$	Electrode
	$\nabla \cdot (-\sigma_{e^{-}}^{\mathrm{eff}}\nabla \phi_{e^{-}})=0$	Current collector, Interconnect
Oxygen ion	$\nabla \cdot \left(-\sigma_{O^{2-}}^{\mathrm{eff}}\nabla \phi_{O^{2-}}\right)=S^{\mathrm{eff}}\;i$	Electrode
	$\nabla \cdot (-\sigma_{O^{2-}}^{\mathrm{eff}}\nabla \phi_{O^{2-}})=0$	Electrolyte, Interlayer
Mass	$\frac{\partial \left(\varepsilon_{p}\rho \right)}{\partial t}+\nabla \cdot \left(\rho u\right)\;=\;\sum {\dot{s}}_{i}$	Current collector, Electrode, Fluid channel
Momentum	$\frac{\partial (\rho u)}{\partial t}+\left(\frac{\rho }{\varepsilon_{p}}u\cdot \nabla \right)u=-\varepsilon_{p}\nabla p+\nabla \cdot \left[\mu \{\nabla u+{\left(\nabla u\right)}^{T}-\frac{2}{3}\left(\nabla \cdot u\right)I\}\right]-\frac{\mu }{\zeta }u$	Current collector, Electrode
	$\frac{\partial (\rho u)}{\partial t}+\left(\frac{\rho }{\varepsilon_{p}}u\cdot \nabla \right)u=-\varepsilon_{p}\nabla p+\nabla \cdot \left[\mu \{\nabla u+{\left(\nabla u\right)}^{T}-\frac{2}{3}\left(\nabla \cdot u\right)I\}\right]$	Fluid channel
Species	$\frac{\partial \left(\varepsilon_{p}\rho \omega_{i}\right)}{\partial t}+\nabla \cdot \left(\rho \omega_{i}u\right)\;=\nabla \cdot \left(\rho D_{i}^{\mathrm{eff}}\nabla \omega_{i}\right)+{\dot{s}}_{i}$	Current collector, Electrode, Fluid channel
Energy	${\left(\rho c_{p}\right)}^{\mathrm{eff}}\frac{\partial T}{\partial t}+\rho c_{p}u\cdot \nabla T=\nabla \cdot \left(\lambda^{\mathrm{eff}}\nabla T\right)-\sum c_{p,i}\nabla T\cdot J_{i}+\sigma_{e^{-}}^{\mathrm{eff}}{\left(\nabla \phi_{e^{-}}\right)}^{2}+\sigma_{O^{2-}}^{\mathrm{eff}}{\left(\nabla \phi_{O^{2-}}\right)}^{2}+S^{\mathrm{eff}}i\left(\eta_{\mathrm{act}}+T\frac{\partial \Delta \phi^{\mathrm{eq}}}{\partial T}\right)$	Electrode
	${\left(\rho c_{p}\right)}^{\mathrm{eff}}\frac{\partial T}{\partial t}+\rho c_{p}u\cdot \nabla T=\nabla \cdot \left(\lambda^{\mathrm{eff}}\nabla T\right)-\sum c_{p,i}\nabla T\cdot J_{i}+\sigma_{e^{-}}^{\mathrm{eff}}{\left(\nabla \phi_{e^{-}}\right)}^{2}$	Interconnect, Current collector, Fluid channel
	${\left(\rho c_{p}\right)}^{\mathrm{eff}}\frac{\partial T}{\partial t}+\rho c_{p}u\cdot \nabla T=\nabla \cdot \left(\lambda^{\mathrm{eff}}\nabla T\right)-\sum c_{p,i}\nabla T\cdot J_{i}+\sigma_{O^{2-}}^{\mathrm{eff}}{\left(\nabla \phi_{O^{2-}}\right)}^{2}$	Electrolyte, Interlayer
	${\left(\rho c_{p}\right)}^{\mathrm{eff}}\frac{\partial T}{\partial t}+\rho c_{p}u\cdot \nabla T=\nabla \cdot \left(\lambda^{\mathrm{eff}}\nabla T\right)-\sum c_{p,i}\nabla T\cdot J_{i}$	Sealant

**Table 3:** Constitutive equations.

Variable	Constitutive equation	Domain
Effective electronic conductivity	$\;\sigma_{e^{-}\;}^{\mathrm{eff}}=P_{e^{-}}\;\frac{\varepsilon_{e^{-}}}{\tau_{e^{-}}}\sigma_{e^{-}}$	Electrode, Interconnect
	$\;\sigma_{e^{-}\;}^{\mathrm{eff}}=\left(1-\varepsilon_{p}\right)\;\sigma_{e^{-}}$	Current collector
Effective ionic conductivity	$\;\sigma_{O^{2-}\;}^{\mathrm{eff}}=P_{O^{2-}}\;\frac{\varepsilon_{O^{2-}}}{\tau_{O^{2-}}}\sigma_{O^{2-}}$	Electrode, Electrolyte, Interlayer
Equilibrium electric potential difference	$\Delta \;\phi_{a}^{\mathrm{eq}}=\frac{\left({\bar{\mu }}_{H_{2}O}^{\circ }-{\bar{\mu }}_{H_{2}}^{\circ }-{\bar{\mu }}_{O^{2-}}^{\circ }\right)}{2F}+\frac{RT}{2F}\ln\frac{p_{H_{2}O}}{p_{H_{2}}}$ $\Delta \;\phi_{c}^{\mathrm{eq}}=\frac{\left({\bar{\mu }}_{O_{2}}^{\circ }-2{\bar{\mu }}_{O^{2-}}^{\circ }\right)}{4F}+\frac{RT}{4F}\ln\frac{p_{O_{2}}}{p^{0}}$	
Effective reaction site density	*S* ^eff^ = *S*∏*P_i_*	
Mixture dynamic viscosity	µ = ∑ω_*i*_µ_*i*_	
Binary diffusivity	$D_{ij}=\frac{3.16\times 10^{-4}T^{1.75}{\left(M_{i}^{-1}+M_{j}^{-1}\right)}^{1/2}}{p{\left[{\left(\Sigma_{i}\right)}^{1/3}+{\left(\Sigma_{j}\right)}^{1/3}\right]}^{2}}$	
Knudsen diffusivity	$D_{\mathrm{Kn},i}=\frac{d_{p}}{3}\sqrt{\frac{8RT}{\pi M_{i}}}$	
Mixture averaged diffusivity	$D_{\mathrm{mix},i}=\frac{1-x_{i}}{\sum_{j\neq i}^{N}\frac{x_{j}}{D_{ij}}}$	
Effective diffusivity	$\;D_{i}^{\mathrm{eff}}=P_{p}\;\frac{\varepsilon_{p}}{\tau_{p}}{\left(\frac{1}{D_{\mathrm{Kn},i}}+\frac{1}{D_{\mathrm{mix},i}}\right)}^{-1}$	Electrode
	$\;D_{i}^{\mathrm{eff}}=\varepsilon_{p}\;D_{\mathrm{mix},i}$	Current collector, Fluid channel
Gas permeability	$\zeta =\frac{{\varepsilon_{p}}^{3}}{{\left(1-\varepsilon_{p}\right)}^{2}}\;\frac{d_{s}^{2}}{72\tau_{p}}$	Electrode
Effective thermal conductivity	λ^eff^ = ε_p_ λ_f_ + (1 − ε_p_)λ_s_	Current collector, Electrode, Fluid channel
	λ^eff^ = λ_s_	Interconnect, Sealant
	where λ_f_ = ∑*x_i_* λ_*i*_ and $\lambda_{s}=\prod {\lambda_{i}}^{\frac{\varepsilon_{i}}{1-\varepsilon_{p}}}\;$	
Mixture‐specific heat capacity	$\;{\left(\rho c_{p}\right)}^{\mathrm{eff}}=\frac{{\left(\rho c_{p}\right)}_{f}^{\mathrm{eff}}+{\left(\rho c_{p}\right)}_{s}^{\mathrm{eff}}}{2}$	Fluid channel
	$\;{\left(\rho c_{p}\right)}^{\mathrm{eff}}={\left(\rho c_{p}\right)}_{s}^{\mathrm{eff}}$	Interconnect, Sealant
	(ρ*c*_p_)^eff^ = ε_p_ (ρ*c*_p_)_f_ + (1 − ε_p_)(ρ*c*_p_)_s_	Current collector, Electrode
	where (ρ*c*_p_)_f_ = ∑ρ_*i*_ *c*_p,*i*_ and (ρ*c*_p_)_s_ = ρ_s_ *c*_s_	

The computational domain (Figure 4a) represents a 2D single channel in the *xy*‐plane, assuming negligible depth‐wise transport^[^ ^38a,c^ ^]^ and a periodic SOFC structure. This simplification can reduce computational costs while retaining the essential physical behavior. This domain includes the metallic interconnects, porous current collectors, the sealant, and the SOFC. Six conservation equations—electron charge, ion charge, mass, momentum, species, and energy—were solved and coupled with electrochemical reactions. Hydrogen oxidation and oxygen reduction were modeled at Ni–YSZ–pore triple‐phase boundaries (TPBs) and LSC–pore double‐phase boundaries (DPBs), respectively,^[^ ^37^, ^40^ ^]^ using modified nonlinear Butler–Volmer‐type equations.^[^ ^41^ ^]^

(2) $$i=i_{0}\;\left[\exp\left(\frac{2\alpha F\eta_{\mathrm{act}}}{RT}\right)-\exp\left(-\frac{2\left(1-\alpha \right)F\eta_{\mathrm{act}}}{RT}\right)\;\right]$$

where α, *F*, *R*, and *T* are the charge transfer coefficient, Faraday constant, universal gas constant, and absolute temperature, respectively. In addition, η_act_ and η_con_ are the activation and concentration overpotentials, defined as

(3) $$\eta_{\mathrm{act}}=\left(\phi_{e^{-}}-\phi_{O^{2-}}\right)\;-\Delta \phi^{\mathrm{eq}}$$

(4) $$\eta_{\mathrm{con},a}=\frac{RT}{2F}\;\ln\left(\frac{p_{H_{2}}^{\mathrm{bulk}}}{p_{H_{2}}}\frac{p_{H_{2}O}}{p_{H_{2}O}^{\mathrm{bulk}}}\right)$$

(5) $$\eta_{\mathrm{con},c}=\frac{RT}{4F}\;\ln\left(\frac{p_{O_{2}}}{p_{O_{2}}^{\mathrm{bulk}}}\right)$$

Here, $\phi_{e^{-}}$ and $\phi_{O^{2-}}$ are the electric potentials of electrons and ions, respectively; Δϕ^eq^ is the equilibrium electric potential difference; and *p* is the partial pressure. Equation (2) has been widely used in SOFC modeling studies to address topics such as elementary reaction kinetics,^[^ ^42^ ^]^ microstructural effects,^[^ ^43^ ^]^ and commercial‐level stack simulations.^[^ ^44^ ^]^ The exchange current density *i*_0_ depends on the temperature and partial pressure, with the activation energy *E_a_* sourced from the literature.^[^ ^44a^ ^]^ In this model, only the pre‐exponential factor *A* was calibrated to 4.56×10^12^ and 3.26×10^10^ A m^−2^ for the anode and cathode, respectively, to match the experimental results as follows:

(6) $$i_{0,a}=A_{a}\;\frac{{\left(\frac{p_{H_{2}}}{p_{H_{2}}^{\mathrm{eq}}}\right)}^{\frac{1}{4}}\;{\left(\frac{p_{H_{2}O}}{p^{0}}\right)}^{\frac{3}{4}}}{1+{\left(\frac{p_{H_{2}}}{p_{H_{2}}^{\mathrm{eq}}}\right)}^{\frac{1}{2}}}\exp\left(-\frac{E_{a,a}}{RT}\right)$$

(7) $$i_{0,c}=A_{c}\;\frac{{\left(\frac{p_{O_{2}}}{p_{O_{2}}^{\mathrm{eq}}}\right)}^{\frac{1}{4}}\;}{1+{\left(\frac{p_{O_{2}}}{p_{O_{2}}^{\mathrm{eq}}}\right)}^{\frac{1}{2}}}\exp\left(-\frac{E_{a,c}}{RT}\right)$$

The effective conductivities and gas diffusivities were calculated by modifying the bulk transport properties^[^ ^44a^ ^]^ using microstructural parameters derived from the 3D FIB–SEM analysis,^[^ ^39^ ^]^ The gas permeabilities in the porous structures were calculated using the Kozeny–Carman equation,^[^ ^45^ ^]^ except for the anode support, for which a gas permeability of 10^−14^ m^2^ was used.^[^ ^46^ ^]^ Gas mixtures were treated as ideal, with temperature‐dependent properties (e.g., viscosity and specific heat).^[^ ^47^ ^]^ Gas diffusion was modeled using mixture diffusivity as the primary mechanism, with the Knudsen diffusivity included for the porous electrodes.

A steady‐state solution under normal air supply at 0.5 A cm^−2^ was used as the initial condition to simulate air supply interruption (**Table** **4** and Table S5, Supporting Information). An electric current was applied to the top surface of the upper interconnect, while the bottom surface of the lower interconnect was grounded. The flow velocities at the fuel and air inlets matched the experimental volumetric flow rates. The inlet gas compositions were set to 97% H_2_–3% H_2_O and 21% O_2_–79% N_2_ for the fuel and air sides, respectively, with a total pressure of 1 atm. At the fuel outlet, a no‐diffusion flux condition was imposed, while the air outlet composition was fixed to that of external air to account for backflow during interruption. The thermal boundary conditions included fixed gas inlet surfaces at the furnace temperature (700 °C), no conductive flux at the gas outlets, and convective heat flux elsewhere, with a heat transfer coefficient of 10 W m^−2^ K^−1^ to simulate the heat exchange with the surrounding air. The air inlet flux was linearly reduced to zero over 0.1 s, maintaining a constant current load. For *t* > 0.1 s, the oxygen partial pressure in the air‐side domain dropped below 10^−6^ atm, invalidating the continuum assumption. At *t* = 1.12 s, the oxygen partial pressure was exponentially reduced, and the air mass, species, and momentum conservation equations were excluded while the other analyses continued.

**Table 4:** Boundary conditions at the initial steady state.

	Inlet $\;(x=-\frac{L}{2})$		Outlet $\;(x=\frac{L}{2}\;)$		Interconnect		
	Fuel	Air	Fuel	Air	Top	Bottom	Side
Electrical	–		–		$n\cdot \;i_{e^{-}}=\frac{L_{\mathrm{cell}}}{L}\;i_{\mathrm{cell}}$	$\phi_{e^{-}}=0$	$n\cdot \;i_{e^{-}}=0$
Species	ω_*i*_ = ω_*i*,0_		$n\cdot \;(-\rho D_{i}^{\mathrm{eff}}\nabla \omega_{i})=0$	ω_*i*_ = ω_*i*,0_	–		
Momentum	− **n** · **u** = *u*_0_		*p* = *p*_0_		–		
Thermal	*T* = *T*_0_		**n** · (− λ^eff^∇*T*) = 0		− **n** · (− λ^eff^∇*T*) = *h*(*T*_amb_ − *T*)		

Simulations were conducted using COMSOL Multiphysics 6.1,^[^ ^48^ ^]^ and the governing equations were solved via the finite element method using the Direct PARDISO solver.^[^ ^48^ ^]^ A relative tolerance of 10^−4^ ensured convergence, and the backward differentiation formula was used for time‐stepping, with a maximum time step of 10^−2^ s based on the air‐side mass‐transfer timescale (<0.1 s).^[^ ^38^, ^49^ ^]^ The mesh and time‐step independence were verified (Figure S17, Supporting Information). The calculations were performed on a 12th Gen Intel Core i5‐12600K CPU, requiring ≈50 min and utilizing 5 GB of RAM.

### DFT Calculations

Spin‐polarized DFT calculations were performed using the Vienna Ab initio Simulation Package software (VASP 6.4.2) with the projector‐augmented wave (PAW) method.^[^ ^50^ ^]^ A plane‐wave kinetic energy cutoff of 520 eV and a Gaussian smearing of 0.05 eV width were employed. The DFT‐D4 method was implemented to correct Van der Waals interaction.^[^ ^51^ ^]^ The exchange‐correlation energy was treated using Perdew‐Burke‐Ernzerhof (PBE) functional based on the generalized gradient approximation (GGA).^[^ ^52^ ^]^ All geometry optimization was conducted until the energy and force converged to 10^−5^ eV and 0.03 eV Å^−1^, respectively. The DFT+U method was applied for Co 3d state with U_eff_ = 3.35 eV to correct the self‐interaction in DFT calculations.^[^ ^53^ ^]^ A 4 × 4 × 4 and 4 × 4 × 1 Monkhorst‐Pack *k*‐points meshes was adopted for bulk and slab model calculations, respectively.^[^ ^54^ ^]^ A vacuum thickness of at least 10 Å along the c‐axis was used to avoid the interaction between periodic images. Based on the optimized structure of (2 × 2 × 2) LaCoO_3_ (LCO) bulk supercell with a lattice parameter of 7.61 Å (Figure S18, Supporting Information), a eight atomic layers of (2 × 2) LSC (001) slab model was constructed by substituting six La atoms with Sr atoms to consider the stochiometry of experimentally synthesized LSC with a 6:4 = La:Sr ratio. During structural optimization, the four bottom atomic layers of the (001) LSC slab model were fixed in their bulk position, whereas the top four layers were allowed to relax. To elucidate the effect of oxygen vacancies on Sr migration toward the surface in LSC, the segregation energy (E_seg_) was calculated based on pristine LSC and the most stable LSC structure with an oxygen vacancy. The optimized structures of LSC slab models are shown in Figure S19 (Supporting Information). The oxygen vacancy formation energy (E_Vo_) was calculated as follows:$E_{V_{O}}=E_{V_{O},LSC}-E_{pristine,LSC}-\frac{1}{2}E_{O_{2}}$ where E_Vo, LSC_, E_pristine, LSC_, and E_O2_ are the total energies of the LSC with oxygen vacancy, pristine LSC surface slab, and isolated O_2_ molecule, respectively. The E_seg_ was calculated as follows:*E_seg_* = *E*_*Sr* − *surf*, *LSC*_ − *E*_*Sr* − *bulk*, *LSC*_ where E_Sr‐surf, LSC_ and E_Sr‐bulk, LSC_ are the total energies of the LSC slab with Sr exposed on the surface and the LSC slab with Sr located in the bulk, respectively.

## Conflict of Interest

The authors declare no conflict of interest.

## Author Contributions

H.S, J.E.W., W.L. and H.C. equally contributed to this work. S.S. contributed to conceptualization, data curation, formal analysis, methodology, investigation, validation, visualization, writing the original draft, and review and editing. J.W. contributed to methodology, investigation, and writing the original draft. W.L. contributed to investigation, software, and writing the original draft. H.C. contributed to investigation, formal analysis, and writing the original draft. S.P. contributed to investigation. Y.S. contributed to methodology. I.L. contributed to conceptualization and methodology. T.J.L. contributed to software and writing the original draft. K.K. contributed to software and review and editing. J.H. contributed to investigation, software, supervision, writing the original draft, and review and editing. H.J.C. contributed to investigation, formal analysis, writing the original draft, and review and editing. K.J.Y. contributed to conceptualization, methodology, funding acquisition, supervision, writing the original draft, and review and editing.

## Supporting information



Supporting Information

## Data Availability

The data that support the findings of this study are available from the corresponding author upon reasonable request.
