# Supplementary material for: Catastrophic Degradation in Solid Oxide Fuel Cells Caused by Air Supply Interruption in Real‐World Operations: Fundamental Mechanisms and Mitigation Strategies
Source: Adv Sci (Weinh). 2025 Sep 30;13(3):e16807. doi: 10.1002/advs.202516807 (PMC12806198; doi:10.1002/advs.202516807)
Supplement: Supplementary file 1 — Supporting Information [file ADVS-13-e16807-s001.docx]

Supporting Information

Catastrophic degradation in solid oxide fuel cells caused by air supply interruption in real-world operations: Fundamental mechanisms and mitigation strategies

Haewon Seo,^#^ Ji-eun Won,^#^ Wooseok Lee,^#^ Haneul Choi,^#^ Sun-Young Park, Younghun Shin, Insung Lee, Tae Jin Lim, Kyeounghak Kim, Jongsup Hong,* Hye Jung Chang * and Kyung Joong Yoon*

# These authors equally contributed to this work

H. Seo, J. Won, W. Lee, H. Choi, Y. Shin, H. J. Chang, K. J. Yoon

Center for Hydrogen Energy Materials, Korea Institute of Science and Technology, Seoul 02792, Republic of Korea.

E-mail: kjyoon@kist.re.kr, almacore@kist.re.kr

J. Won, W. Lee, J. Hong

School of Mechanical Engineering, Yonsei University, Seoul 03722, Republic of Korea.

E-mail: jongsup.hong@yonsei.ac.kr

S. –Y. Park

Clean Hydrogen Institute-Research Support Department, Korea Institute of Science and Technology, Seoul 02792, Republic of Korea

I. Lee

E&KOA, Daejeon 34325, Republic of Korea.

H. J. Chang

Division of Nano Convergence, KIST School, University of Science and Technology, Seoul 02792, Republic of Korea

E-mail: almacore@kist.re.kr

T. J. Lim, K. Kim

Department of Chemical Engineering, Hanyang University, Seoul 04763, Republic of Korea

**
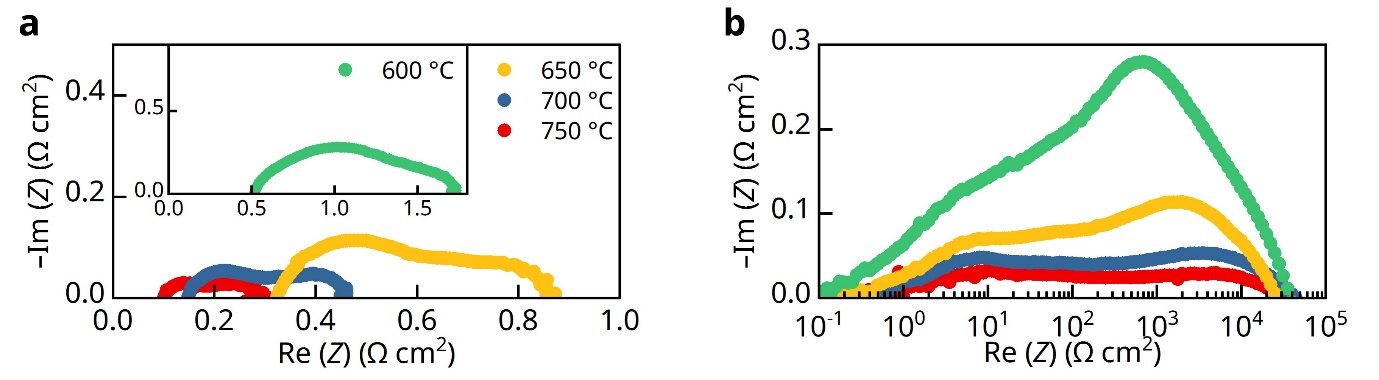
**

**Figure S1.** (a) Nyquist and (b) Bode plots of the EIS spectra for the cell with an LSC cathode, measured in the temperature range of 600–750 °C.

**
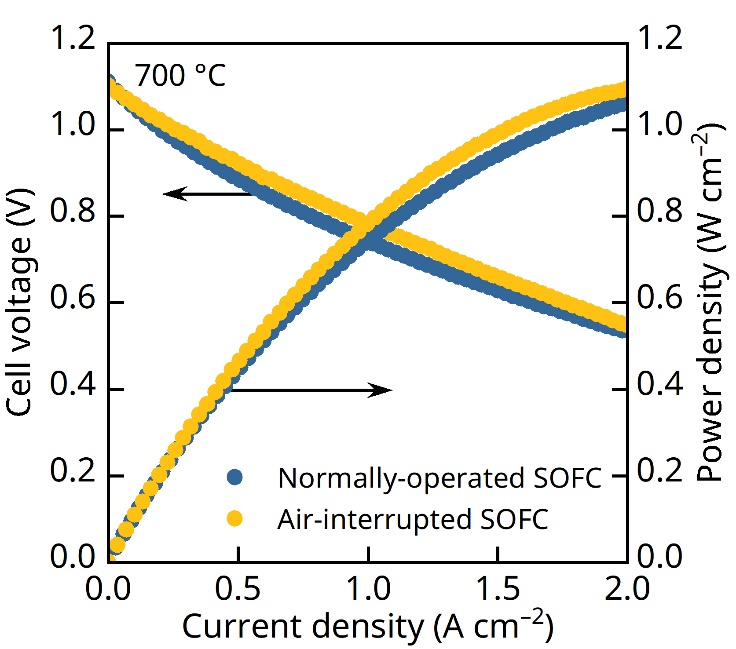
**

**Figure S2.** Comparison of the initial electrochemical performance of two identically fabricated SOFCs with the LSC cathode evaluated at 700 °C with 3% humidified H_2_ as fuel and dry air as the oxidant, showing similar initial performance.


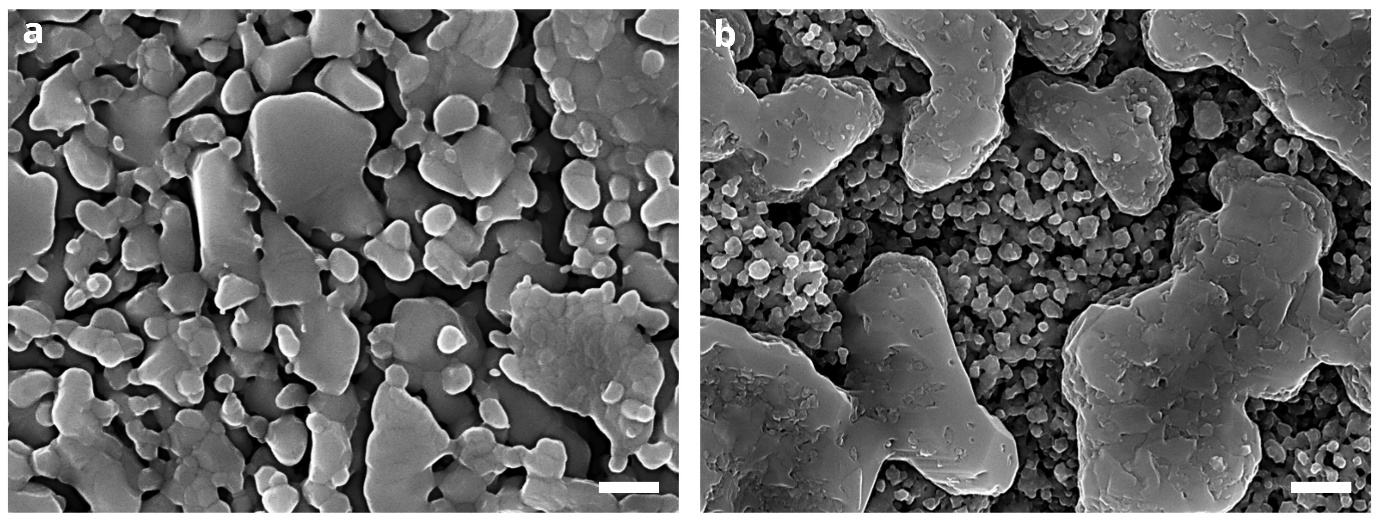


**Figure S3**. SEM images obtained from the top surfaces of the LSC cathodes operated under normal conditions with (a) continuous and (b) interrupted air supplies. The scale bars are 500 nm.


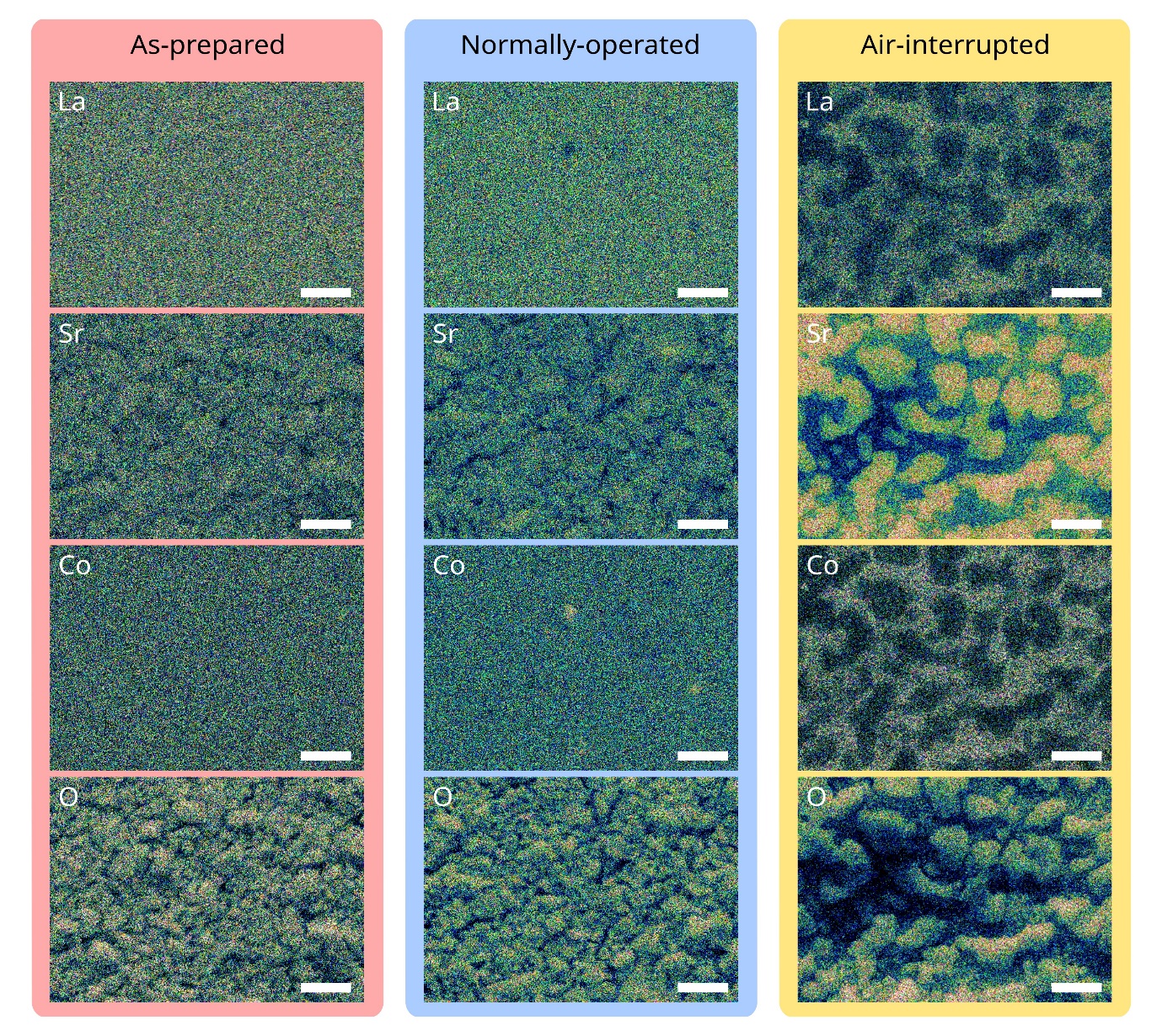
 **Figure S4.** SEM–EDS elemental maps of the top surfaces of the as-fabricated cell (left) and cells operated under normal conditions with continuous (middle) and interrupted (right) air supplies. After the air-supply interruption test, the surface shows pronounced Sr segregation (second row), with Sr forming large coarsened surface precipitates separated from the LSC matrix. Scale bars: 2 µm.


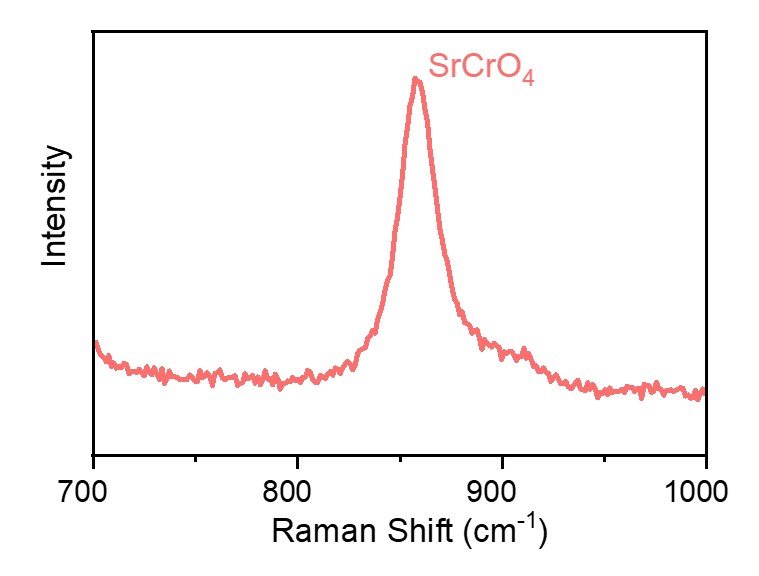


**Figure S5.** Raman spectra of the LSC cathode after air interruption tests, indicating the formation of SrCrO_4_

**
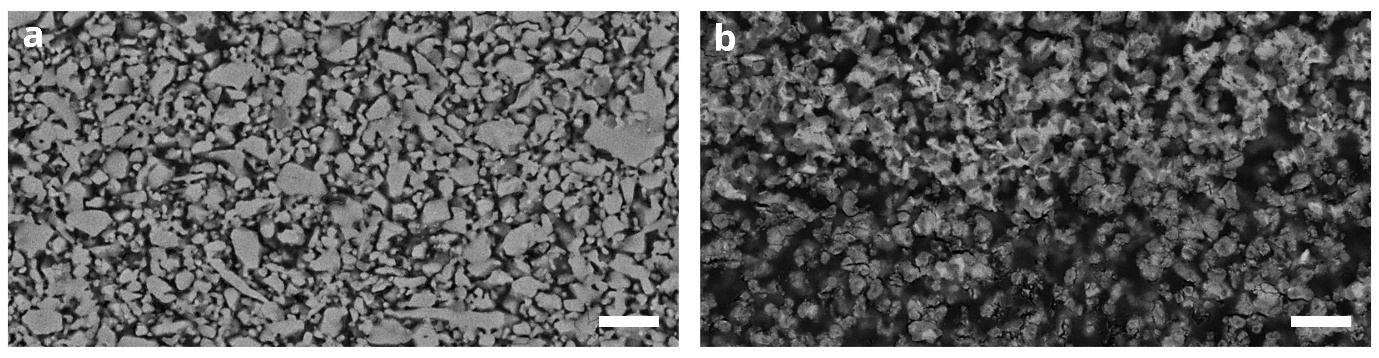
**

**Figure S6.** High-magnification BSE images of the LSC cathodes operated under normal conditions with (a) continuous and (b) interrupted air supplies. The scale bars are 500 nm.


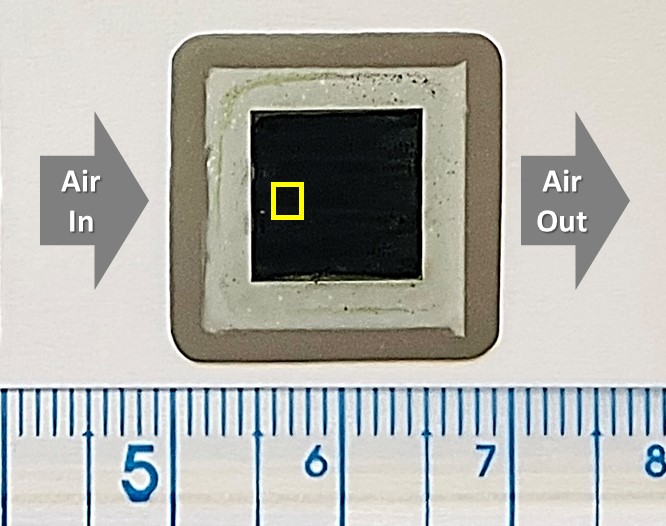


**Figure S7.** Photograph of the cell tested under air-supply interruption, showing the sampling location for STEM analysis highlighted by the yellow square.


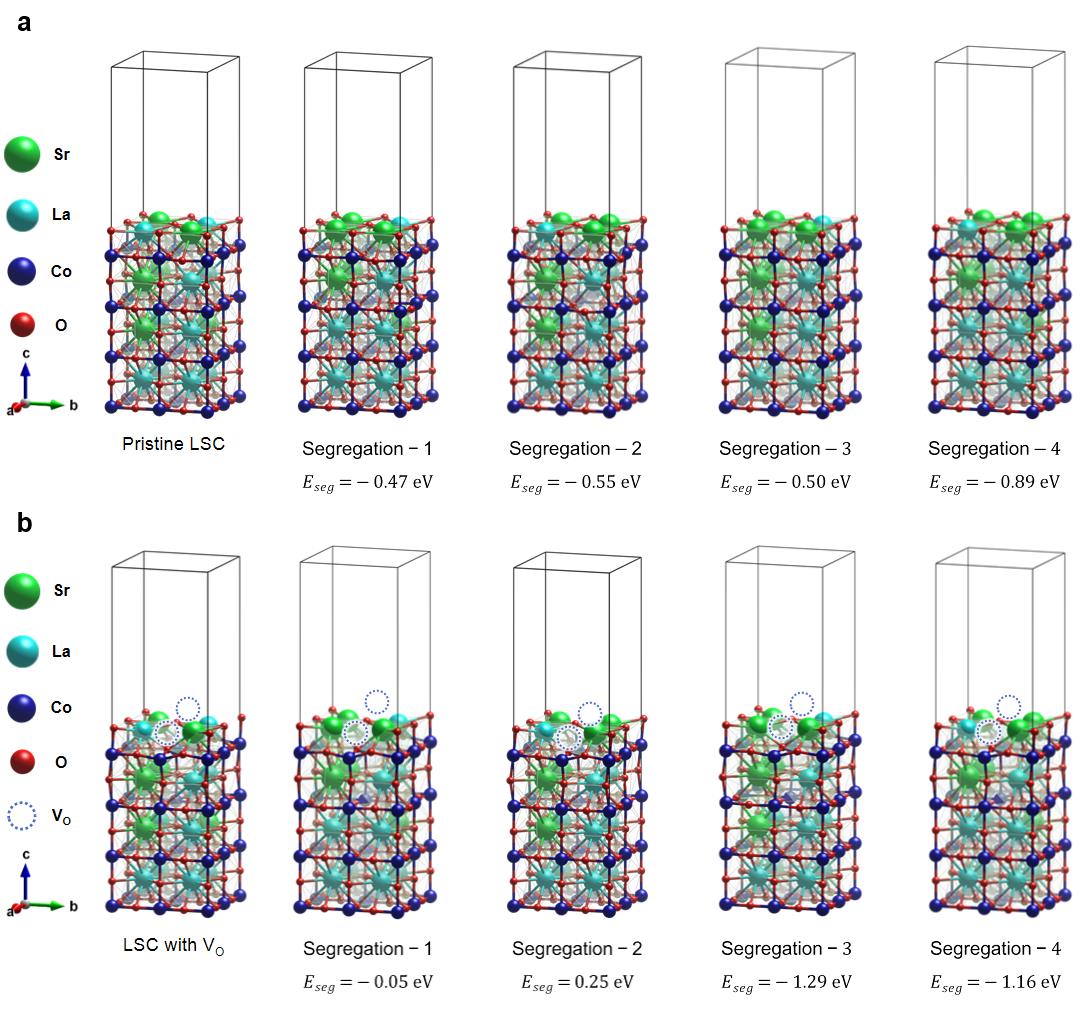


**Figure S8.** Optimized atomic structures used for *E_seg_* calculations: (a) pristine LSC and (b) LSC with a surface oxygen vacancy. Among various atomic and defect configurations, the most thermodynamically stable structure was selected for each case.


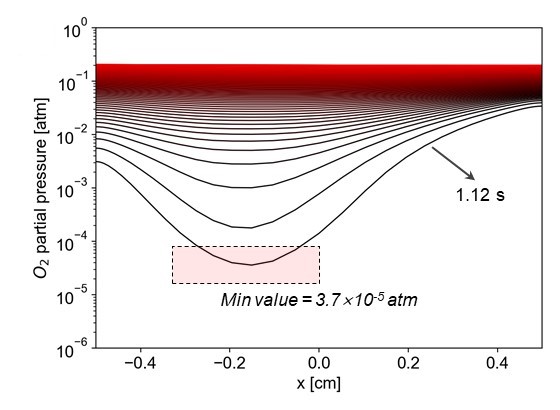


**Figure S9.** Multiphysics modeling of oxygen partial pressure at the outer cathode surface over time during air-supply interruption.


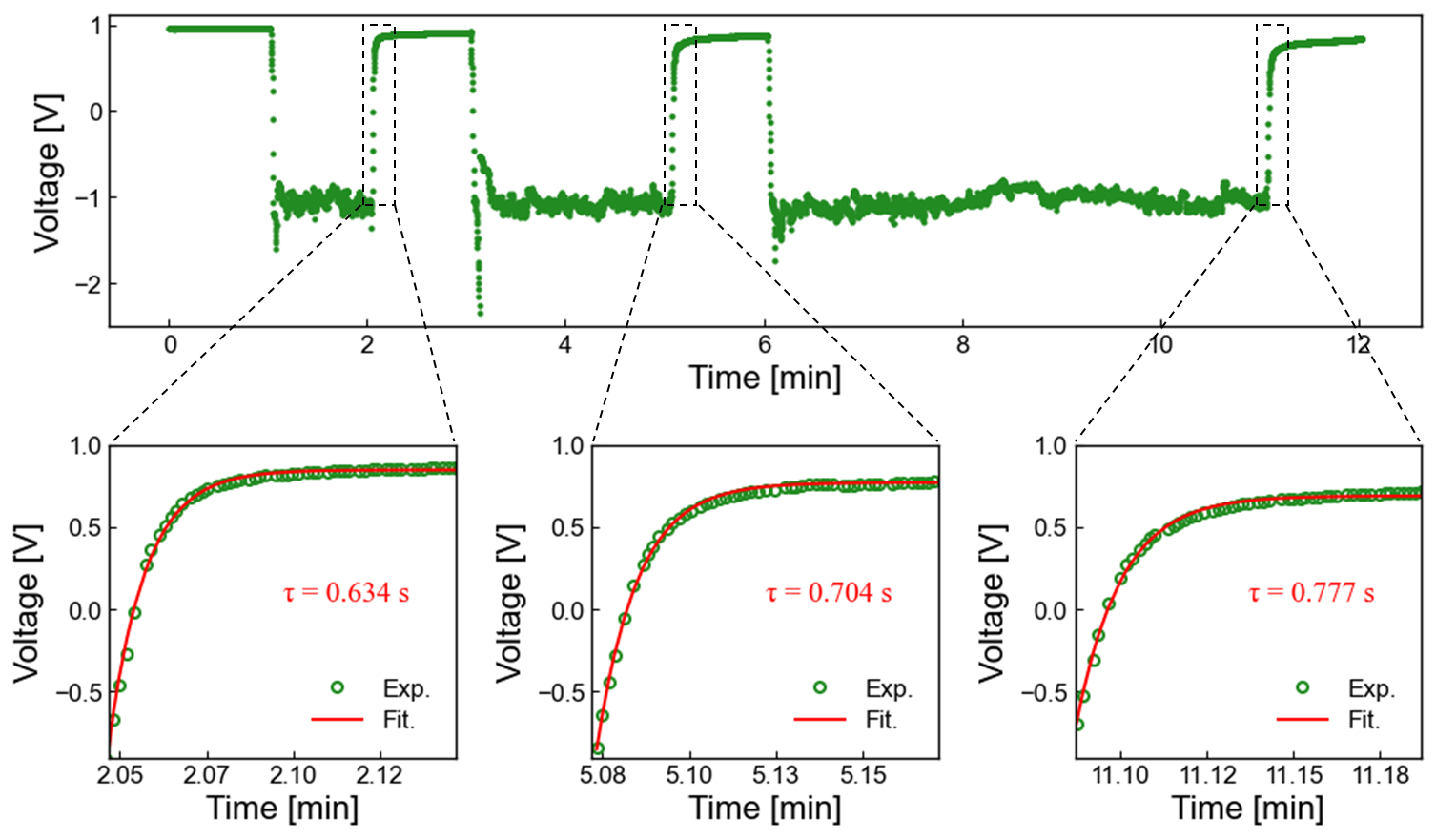


**Figure S10**. Relaxation time analysis of the voltage transition induced by air supply resumption.


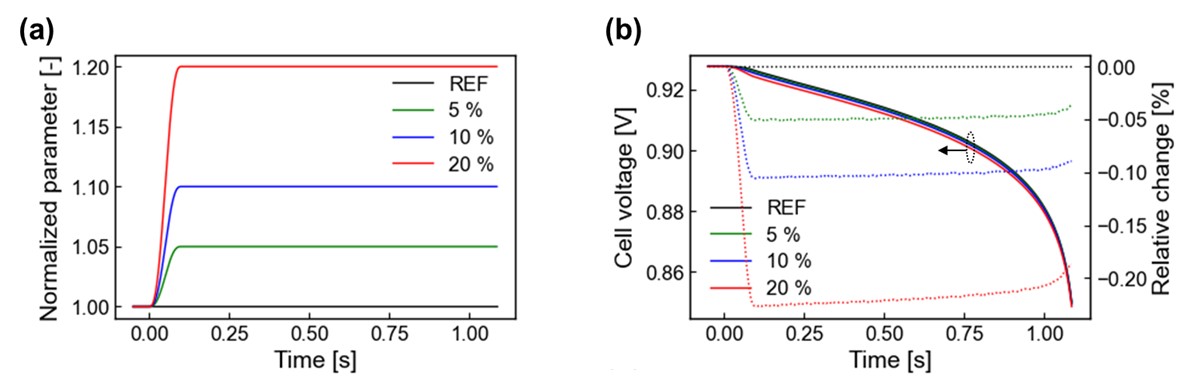


**Figure S11.** Multiphysics simulation results showing the effects of 5%, 10%, and 20% relative variations in porosity and permeability from the reference microstructural parameters. (a) Normalized changes in cathode porosity and permeability. (b) Corresponding changes in cell voltage, with the relative variations from the reference values indicated by dotted lines on the right axis.

**Figure S12.** XRD pattern of LNC powder derived from solution infiltration and calcination at 650 °C.

**
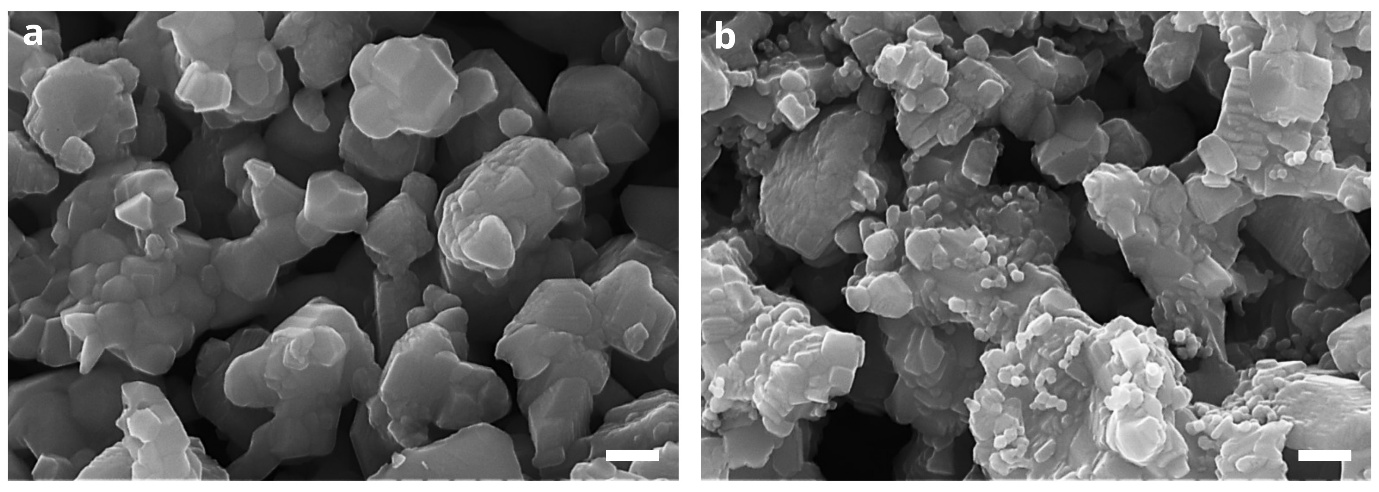
**

**Figure S13.** SEM images of LNO–LNC particles (a) before and (b) after LNC infiltration. The scale bars are 200 nm.

**
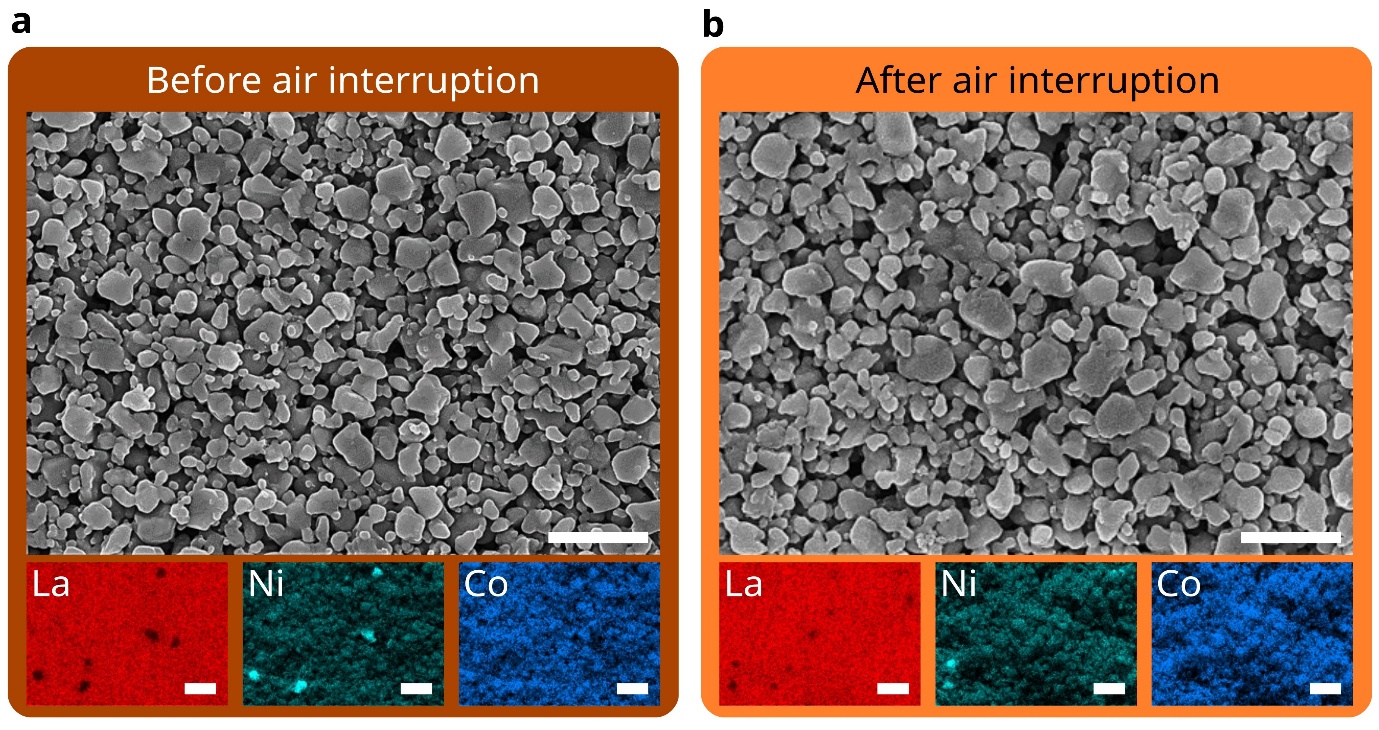
**

**Figure S14.** SEM images and EDS elemental maps obtained from the top surfaces of the cell with the LNC current collector (a) before and (b) after air-supply interruption tests. The scale bars are 2 µm.


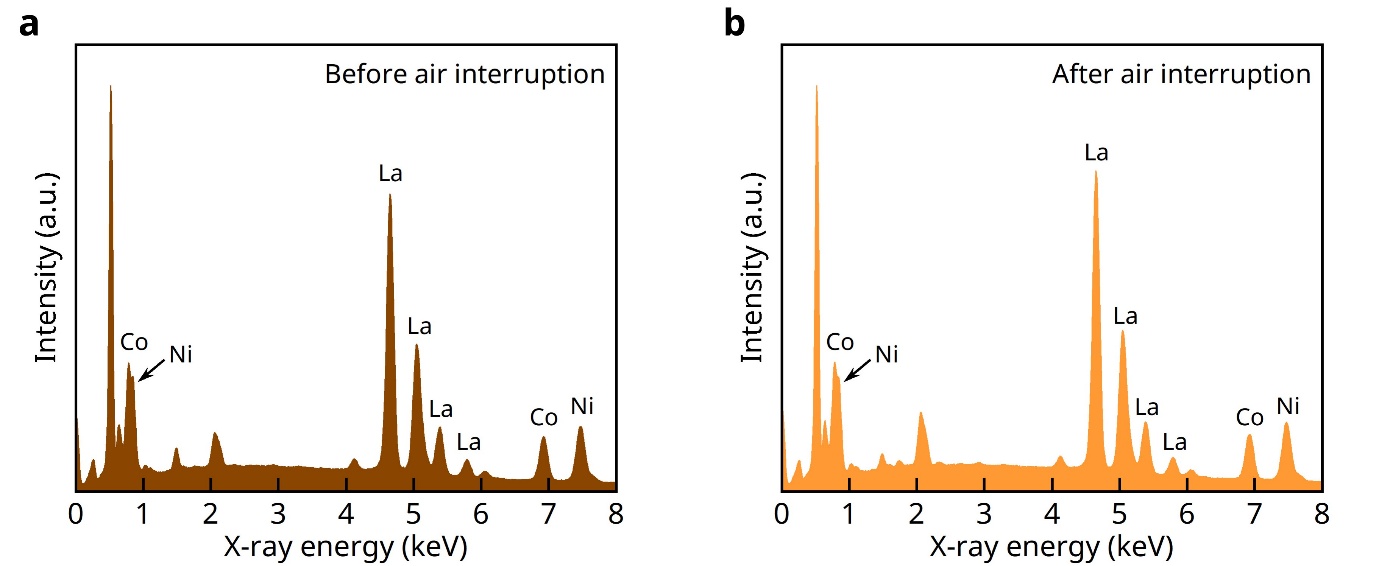


**Figure S15.** EDS spectra obtained from the top surfaces of the cells with the LNC current collector (a) before and (b) after air-supply interruption tests.


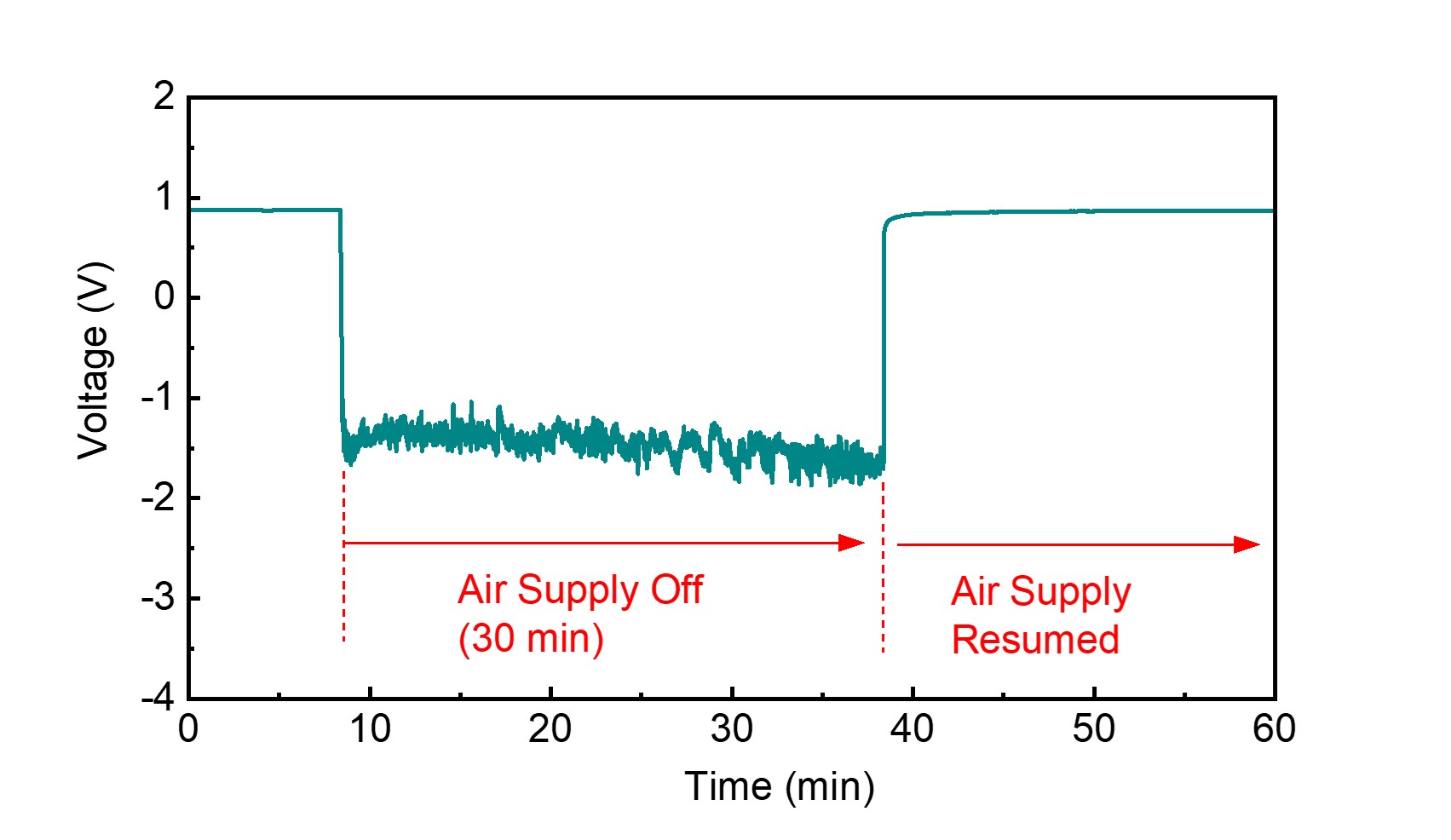


**Figure S16.** Cell voltage response during the extended air interruption test for 30 minutes.


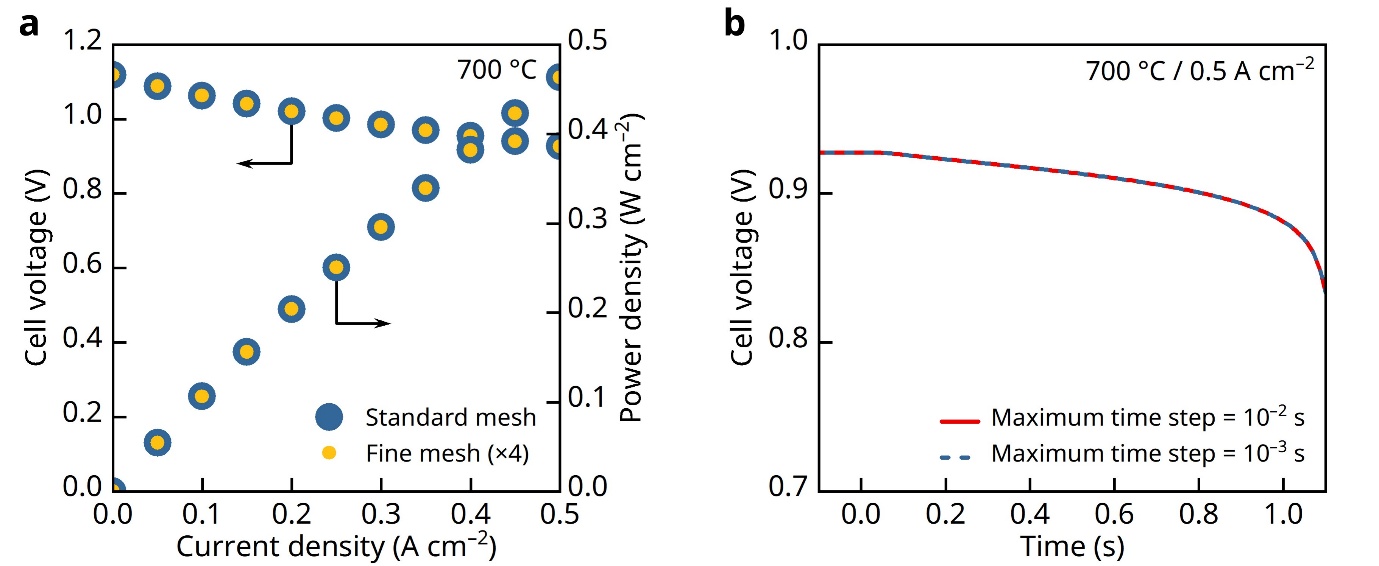


**Figure S17.** Discretization independence test: (a) spatial resolution and (b) temporal resolution.**
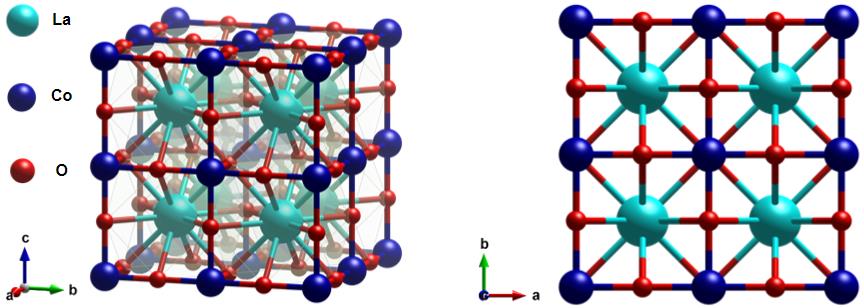

Figure S18**. Size and top views of the optimized structure of (2 × 2 × 2) LCO bulk supercell.

**
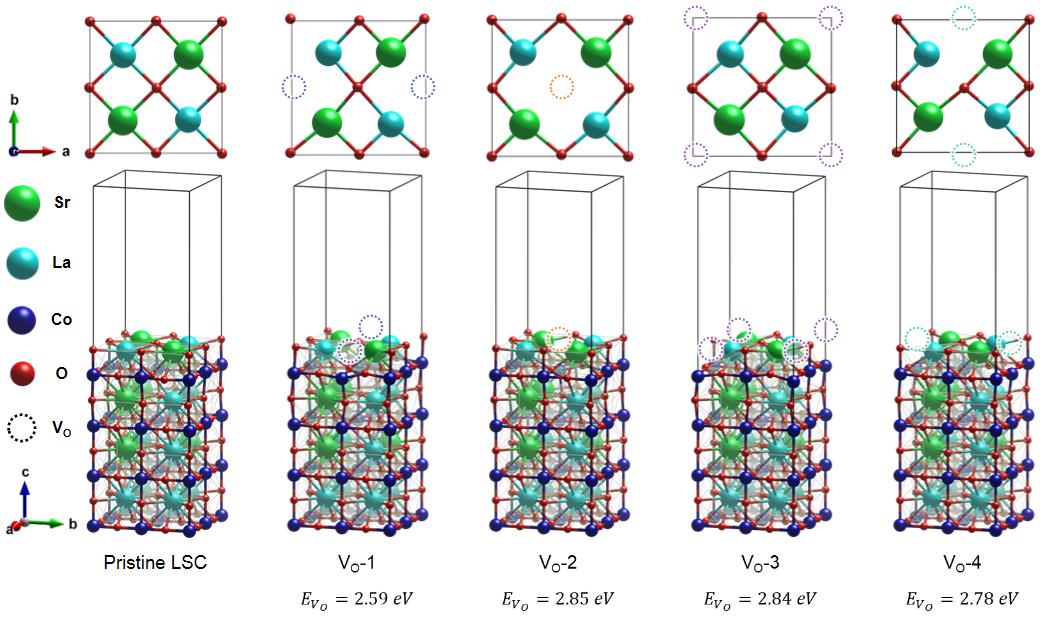
**

**Figure S19.** Considered oxygen vacancy configurations at the surface of LSC(001). We examined four identical surface oxygen vacancy sites considering the symmetry of surface atomic structure and periodic boundary system.

**Table S1.** EDS quantitative compositional analysis results on the cathode surface of the as-fabricated, normally operated and air-interrupted cells.

|  | La | Sr | Co | O | Cr |
| --- | --- | --- | --- | --- | --- |
| As-prepared | 11.9 | 6.83 | 18.0 | 63.2 | - |
| Normally operated | 12.2 | 6.92 | 18.6 | 62.0 | 0.28 |
| Air-interrupted | 1.56 | 11.7 | 2.23 | 76.2 | 8.35 |

**Table S2.** Geometric dimensions.

| Component | Height (mm) | Width (mm) |
| --- | --- | --- |
| Interconnect | 10 | 30 |
| Fluid channel | 1.5 | 30 |
| Current collector (cathode) | 0.3 | 10 |
| Current collector (anode) | 1.2 | 20 |
| Cathode | 0.030 | 10 |
| Interlayer | 0.0030 | 20 |
| Electrolyte | 0.0040 | 20 |
| AFL | 0.012 | 20 |
| ASL | 0.40 | 20 |

**Table S3.** Material properties.

| Property | Material | Value |
| --- | --- | --- |
| Electrical conductivity  (S m^–1^) | Ni | $\sigma_{e^{-},\mathrm{Ni}}=3.27\times{10}^{6}-1065.3T$ |
|  | LSC | $\sigma_{e^{-},\mathrm{LSC}}=4.86\times{10}^{5}-298T$ |
|  | Au | $\sigma_{e^{-},\mathrm{Au}}=4.5\times{10}^{7}$ |
|  | SUS | $\sigma_{e^{-},\mathrm{SUS}}=8.7\times{10}^{5}$ |
| Ionic conductivity  (S m^–1^) | YSZ | $\sigma_{O^{2-},\mathrm{YSZ}}=3.34\times{10}^{4}\exp\left( -\frac{{8.56\times10}^{4}}{RT} \right)$ |
|  | GDC | $\sigma_{O^{2-},\mathrm{GDC}}=\frac{1.09\times{10}^{7}}{T}\exp\left( -\frac{{6.17\times10}^{4}}{RT} \right)$ |
|  | LSC | $\sigma_{O^{2-},\mathrm{LSC}}=1.31\times{10}^{6}\exp\left( -\frac{{9.37\times10}^{4}}{RT} \right)$ |
| Thermal conductivity  (W m^–1^ K^–1^) | Ni | $\lambda_{\mathrm{Ni}}=66.2$ |
|  | YSZ | $\lambda_{\mathrm{YSZ}}=2.1$ |
|  | GDC | $\lambda_{\mathrm{GDC}}=0.98$ |
|  | LSC | $\lambda_{\mathrm{LSC}}=3.94$ |
|  | Au | $\lambda_{\mathrm{Au}}=315$ |
|  | SUS | $\lambda_{\mathrm{SUS}}=20.5$ |
|  | Sealant | $\lambda_{\mathrm{Sealant}}=0.85+5.1$ $\times{10}^{-4}T$ |

**Table S4.** Microstructural parameters

|  | ASL | | | AFL | | | Interlayer | | Cathode | |
| --- | --- | --- | --- | --- | --- | --- | --- | --- | --- | --- |
|  | Ni | YSZ | Pore | Ni | YSZ | Pore | GDC | Pore | LSC | Pore |
| Volume fraction  (%) | 29.9 | 24.5 | 45.6 | 42.1 | 35.3 | 22.7 | 75.6 | 24.4 | 57.0 | 43.0 |
| Mean size  (µm) | 0.94 | 1.07 | 0.85 | 0.78 | 0.84 | 0.72 | 0.78 | 0.39 | 0.67 | 0.65 |
| Tortuosity factor  (–) | 3.34 | 7.15 | 9.20 | 2.60 | 3.38 | 33.4 | 1.22 | 3.70 | 2.31 | 1.81 |
| Connectivity  (%) | 99.7 | 98.2 | 97.2 | 99.9 | 99.7 | 90.8 | 99.9 | 97.7 | 99.9 | 99.9 |
| Reaction site density  (m^–1^) | 5.10×10^2^ | | | 1.18×10^3^ | | | – | | 4.15×10^6^ | |

**Table S5.** Operating conditions under initial steady state (the operating pressure is 1 atm).

| Operating parameter |  | Value |
| --- | --- | --- |
| Cell current density |  | 0.1–1.0 A cm^–2^ |
| Inlet flow velocity | Fuel | 0.82 m s^–1^ |
|  | Air | 0.79 m s^–1^ |
| Inlet gas composition | Fuel | H_2_/H_2_O = 97:3 |
|  | Air | O_2_/N_2_ = 21:79 |
| Inlet gas temperature |  | 700 °C |
| Ambient temperature |  | 700 °C |

**Nomenclature**

$A$ pre-exponential factor (A m^–2^)

$c_{p}$ specific heat capacity (J kg^–1^ K^–1^)

$D_{i}$ diffusion coefficient of species *i* (m^2^ s^–1^)

$E_{a}$ activation energy (kJ mol^–1^)

$F$ Faraday constant (C mol^–1^)

$h$ heat transfer coefficient (W m^–2^ K^–1^)

$\mathbf{I}$ Identity tensor (–)

$\mathbf{i}$ current density (A m^–2^)

$i_{\mathrm{cell}}$ cell current density (A m^–2^)

$i_{0}$ exchange current density (A m^–2^)

$\mathbf{J}_{i}$ diffusion flux vector of species *i* (kg m^2^ s^–1^)

$L$ total length (m)

$L_{\mathrm{cell}}$ cell length (m)

$M_{i}$ molecular weight of species *i* (kg mol^–1^)

$\mathbf{n}$ normal outward unit vector (–)

$P$ connectivity (–)

$p$ partial pressure (–)

$R$ gas constant (J mol^–1^ K^–1^)

$S$ reaction site area per unit volume (m^2^ m^–3^)

$\dot{s}_{i}$ sink/source term of species *i* (kg m^–3^)

$T$ temperature (K)

$t$ time (s)

$\mathbf{u}$ velocity (m s^–1^)

$V$ cell voltage (V)

$x_{i}$ molar fraction of species *i* (–)

**Greek symbols**

$\alpha$ charge transfer coefficient (–)

$\varepsilon_{i}$ volume fraction of phase *i* (–)

$\zeta$ gas permeability (m^2^)

$\eta$ overpotential (V)

$\lambda$ thermal conductivity (W m^–1^ K^–1^)

$\mu$ dynamic viscosity (Pa s)

$\bar{\mu}_{i}^{\circ}$ standard chemical potential of species *i* (kJ mol^–1^)

$\rho$ density (kg m^–3^)

$\Sigma_{i}$ diffusion volume of species *i* (s^4.5^ K^2.625^ mol^0.75^ kg^–2.25^ m^–1.5^)

$\sigma$ electrical conductivity (S m^–1^)

$\tau_{i}$ tortuosity factor of phase *i* (–)

$\phi$ electric potential (V)

$\omega_{i}$ mass fraction of species *i* (–)

**Subscripts**

act activation

a anode

c cathode

conc concentration

e^–^ electronic

O^2–^ ionic

ohm ohmic

f fluid

p pore

s solid

**Superscripts**

eff effective

eq equilibrium

0 standard
